# Applying the Crystalline Sponge Method to Agrochemicals: Obtaining X-ray Structures of the Fungicide Metalaxyl-M and Herbicide *S*-Metolachlor

**DOI:** 10.1021/acs.cgd.1c00196

**Published:** 2021-04-13

**Authors:** Richard D. J. Lunn, Derek A. Tocher, Philip J. Sidebottom, Mark G. Montgomery, Adam C. Keates, Claire J. Carmalt

**Affiliations:** † University College London, Department of Chemistry, 20 Gordon Street, London WC1H 0AJ, U.K.; ‡ Syngenta, Jealott’s Hill International Research Centre, Bracknell, Berkshire RG42 6EY, U.K.

## Abstract

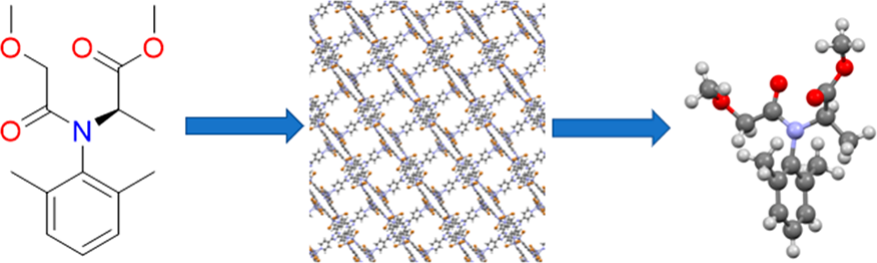

The crystalline sponge method is a technique that provides the ability to elucidate the absolute structure of noncrystalline or hard to crystallize compounds through single-crystal X-ray diffraction by removing the need to obtain crystals of the target compound. In this study the crystalline sponges {[(ZnX_2_)_3_(2,4,6-tris(4-pyridyl)-1,3,5-trazine)_2_].*x*(solvent)}*_n_* (X = I, Br) were used to obtain X-ray structures of the agrochemical active ingredients metalaxyl-M and S-metolachlor. The effect of the temperature used during guest uptake and the influence of changing the host framework ZnX_2_ nodes on guest encapsulation were investigated. Additionally, three compounds containing chemical fragments similar to those of metalaxyl-M and S-metolachlor (phenylacetaldehyde, *N*-ethyl-*o*-toluidine, and methyl phenylacetate) were also encapsulated. This allowed for the effect of guest size on the position that guests occupy within the host frameworks to be examined. The disorder experienced by the guest compounds was documented, and an analysis of the intermolecular host–guest interactions (CH···π and π ···π) used for guest ordering within the host frameworks was also undertaken in this study.

## Abstract 2

The inclusion of five target compounds into the crystalline sponges {[(ZnX_2_)_3_(2,4,6-tris(4-pyridyl)-1,3,5-trazine)_2_].*x*(solvent)}*_n_* (X = I, Br) producing novel inclusion complexes is reported. The target compounds included the first agrochemical fungicide active ingredient (metalaxyl-M) to have their X-ray structure determined by the crystalline sponge method. The influence of guest size, inclusion temperature, and the change in the ZnX_2_ nodes on the positions of the host pores the guests occupy were also analyzed.

## Introduction

The ability to determine the absolute structure of chemical compounds is essential for the progression of chemical research. Traditional methods of characterization such as nuclear magnetic resonance (NMR) spectroscopy, infrared spectroscopy, and mass spectrometry routinely provide the basic structure of a target compound. The determination of the absolute stereochemical structure requires the use of a different technique: e.g., single-crystal X-ray diffraction (SCXRD). The requirement for the sample to be a single crystal is a fundamental limitation of the SCXRD technique; the formation of good-quality single crystals can be a difficult and time-consuming process and is not always possible for all liquid, powder, and amorphous solid compounds.

A procedure that had claimed to overcome this limitation was reported in 2013 by Fujita et al.^1^ The technique, known as the crystalline sponge method (CSM), involves soaking presynthesized crystals of a metal–organic framework (MOF) in a solution of the target compound (referred to as the guest after encapsulation into the MOF pores). Intermolecular interactions between the host MOF and guest compound allow for the guest to become ordered within the pores of the host, resulting in the postcrystallization of the guest within the host framework.^2^ The MOF crystal, with guest encapsulated, can then be analyzed by SCXRD to elucidate the structure of the guest molecule. Due care and attention must be paid to the quality of the crystallographic data obtained. This was highlighted by Fujita et al. in their attempts to assign the absolute stereochemistry of miyakosyne A. After “ambiguities in the crystallographic data” were found, a correction had to be published.^3^ The success of this technique has been demonstrated by the ability of the CSM to determine a variety of structures such as ozonides,^4^ volatile aromatic isomers such as *cis*- and *trans-*asarone,^5^ druglike nucleophilic compounds,^6^ natural products,^7,8^ and metabolites.^9,10^ The CSM has also been used in several mechanistic studies by elucidating the structures of reaction intermediates. If the reaction intermediates match those in a speculated reaction mechanism, they could be used as confirmatory evidence.^11−15^ An example of this is the confirmation of the *syn*-addition mechanism for metal-free diboration.^13^

The most widely used MOF for the CSM is {[(ZnI_2_)_3_(TPT)_2_].*x*(solvent)}*_n_* (**1**; TPT = 2,4,6-tris(4-pyridyl)-1,3,5-trazine).^16^ The organic linker molecule, TPT, is highly aromatic and electron deficient. This allows for the formation of intermolecular π···π and CH···π interactions between the host and electron-rich guest molecules.^17^ The intermolecular interactions formed allow for the guest molecules to become regularly ordered within the MOF pores. The size of the guest molecules that can be encapsulated within the MOF is determined by its pore size; in this case **1** has a pore size of 8 × 5 Å^2^.^18^ Clardy et al. investigated different variants of MOF **1**, {[(ZnX_2_)_3_(TPT)_2_].*x*(solvent)}*_n_* (X = Br (**2**), Cl (**3**)), for use in the CSM. These MOF variants displayed a reduction in the relative scattering contribution from the host framework which allowed for more guest and solvent electron density peaks to be observed.^19^ It has also been noted in other studies that these MOF variants are toward nucleophilic compounds more robust than **1**; this is due to the electron-withdrawing nature of the Br and Cl atoms, which strengthens the pyridyl–zinc bond.^6^ The stronger pyridyl–zinc bond reduces the ability of nucleophilic compounds to exchange with the TPT linker molecule through bonding to the ZnX_2_ nodes.^6^

Though these MOFs have been highly successful in the CSM so far, they do have some limitations that must be taken into account. First, these MOFs are only stable with hydrophobic solvents; contact with hydrophilic solvents will damage the MOF crystals and potentially destroy the single crystallinity. This limits the number of compounds that can be analyzed by this method to those soluble in hydrophobic solvents. For example, Fujita et al. were unable to determine the crystal structures of acyclovir, propranolol, and l-adrenaline when they analyzed nitrogen-containing nucleophilic compounds, as these compounds were insoluble in the solvents that are compatible with the MOF crystals.^6^ Second, the compounds that can be analyzed using these MOFs are limited by the size of the MOF pores (8 × 5 Å^2^ for **1**).^18^ Fujita et al. recommended that the maximum molecular weight for encapsulation into **1** is 500 amu.^20^

The host–guest intermolecular interactions that can be formed to facilitate the ordering of the guest molecules within the MOF pores are mainly dictated by the identity of the MOF organic linker. Studies reported previously have shown that the dominant host–guest interactions formed for ordering guest molecules within the pores of **1** and **2** are CH···π and π···π interactions; this is due to the aromatic and electron-deficient nature of the TPT linker.^17^ Guest ordering using CH···π and π···π interactions has led to disorder being present in some of the guest molecules, increasing the difficulty of structure refinement.^17,21,22^ An example of this is the encapsulation of benzonitrile, where two major cases of disorder were observed. The first rotational disorder was where the phenyl ring position was the same and the nitrile group was oriented in different directions, and the second positional disorder was where the terminal nitrogen atom was common to both components but the phenyl ring was in different positions.^17^ The extent of guest encapsulation is also unpredictable; after a guest inclusion experiment has been performed, it is possible that the guest may not be located within the MOF pores, as the guest has either not entered the pores or is too highly disordered for the structure to be located and refined.

The structural elucidation of new compounds is extremely important during the development of new agrochemical active ingredients. A detailed understanding of the metabolism of the active ingredients is also necessary. This requires a structure elucidation, including if possible the absolute stereochemistry, of all the major metabolites.^23^ The application of the CSM within agrochemical research was demonstrated recently by the successful encapsulation of the herbicide Molinate into the pores of the polar MOF RUM-2; this established the potential of the CSM for structural elucidation in agrochemical research.^24^ Currently there is no single set of experimental conditions that could be used for the encapsulation and structure solution of all potential target compounds via the CSM. Due to the large range of different structures and functionalities that are encountered in agrochemical research,^25,26^ investigation into the optimization of the encapsulation conditions on a range of agrochemicals is required to effectively apply the CSM in this area.

**Figure 1:**
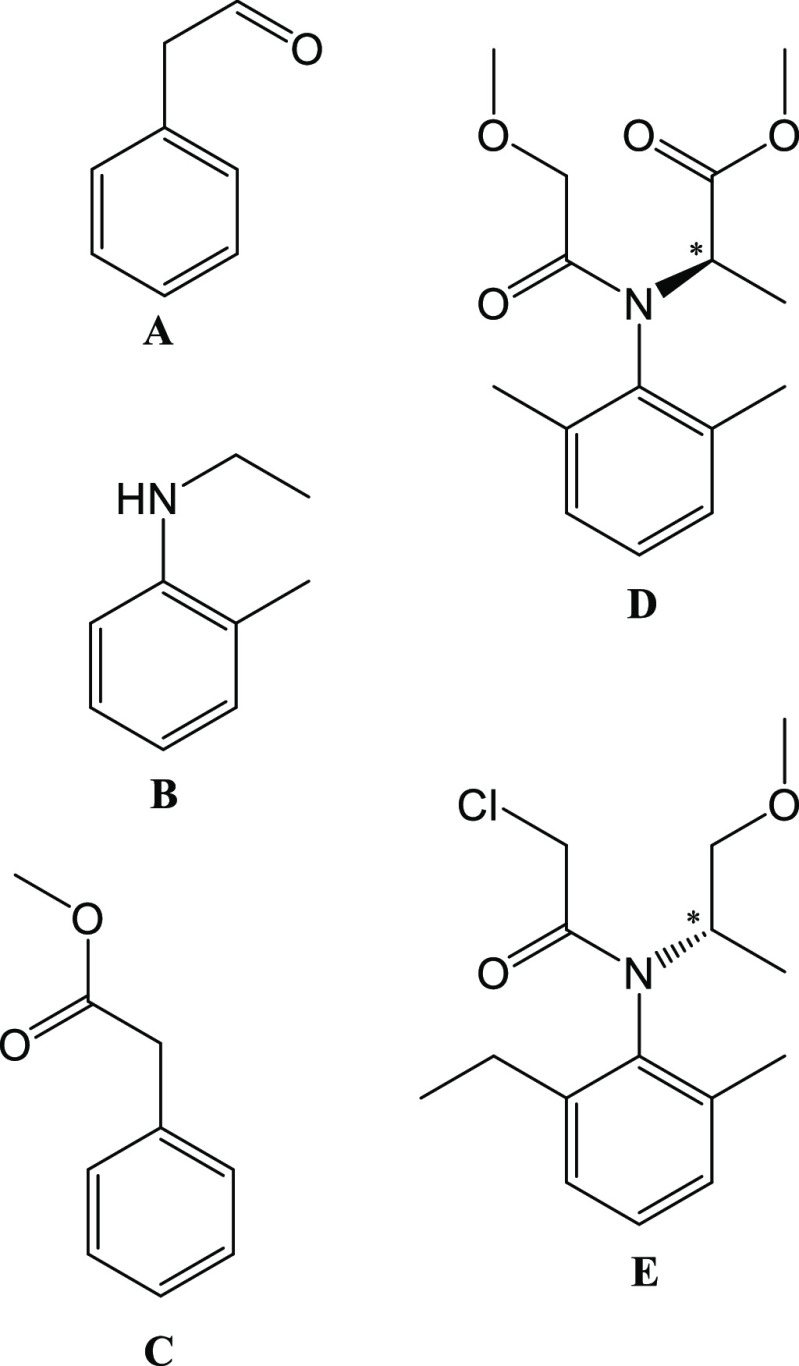
Target compounds chosen for encapsulation into the host MOF **1** and/or **2**: phenylacetaldehyde (**A**), *N*-ethyl-*o*-toluidine (**B**), methyl phenylacetate (**C**), metalaxyl-M (**D**) and S-metolachlor (**E**). An asterisk indicates the guest positions displaying chirality.

In this paper we describe the encapsulation of five new target compounds (Figure 1) into the host frameworks **1** and/or **2**. Three model target compounds were chosen for study, as they exhibit chemical fragments similar to the agrochemicals of interest; these model target compounds were phenylacetaldehyde (**A**), *N*-ethyl-*o*-toluidine (**B**), and methyl phenylacetate (**C**). These compounds allowed for the effect of size on guest position within the host frameworks to be examined. In an expansion of work published previously,^24^ tests were performed using two agrochemicals whose structures are unambiguously known, the fungicide metalaxyl-M^27^ (**D**) and the herbicide S-metolachlor^28,29^ (**E**), with the goal of providing a greater understanding of the encapsulation conditions required for the structural elucidation of agrochemicals.

## Results

Crystals of the host MOFs **1** and **2** were synthesized using procedures based on existing methods in the literature.^19,30^ Once synthesized, the crystals were stored in chloroform at 25 °C in screw-capped vials until required for use in guest encapsulation experiments. Chloroform was chosen as a suitable pore solvent, as all of the target compounds are miscible in chloroform. Also, chloroform is a labile solvent, as only a small number of weak CH···π intermolecular interactions are formed between chloroform molecules and the host frameworks. This helps guest inclusion by allowing a greater quantity of the target compound to enter the MOF pores and reduces the chance of guest molecules occupying the same sites as solvent molecules, which could increase the difficulty of guest structure refinement.^31^

All of the guests used in this investigation are liquid at room temperature; therefore, neat liquids were used during the guest encapsulation experiments. This produced the highest possible concentration gradient in order to ensure the highest possible occupancy of the guest site within the pores of the MOF. Guest encapsulation experiments were performed by submerging host MOF crystals in a small amount of neat guest in a screw-capped vial. These vials were then stored in an incubator maintained at either 25 or 50 °C (Table S1 in the Supporting Information). Temperature-controlled incubators were used to reduce the effect of temperature fluctuations that occur naturally in the laboratory during the day, as temperature fluctuations could cause damage to the host MOF crystals via the formation of small defects. During guest encapsulation the host MOF crystals were regularly checked under a microscope to determine if the crystal quality was being maintained. After the host crystals had been soaking in the guest for a specific length of time (Table S1 in the Supporting Information), an appropriate good-quality single crystal was selected for analysis via SCXRD.

All of the host–guest complexes reported here are novel and were found to maintain the centrosymmetric *C*2/*c* symmetry of the as-synthesized hosts frameworks **1**(1) and **2**(19) and show similar unit cell parameters. The difficulty of guest identification within the host pores varied and was dependent on the guest occupancy. In all cases encapsulated guest molecules that were successfully located were able to be refined with occupancies between 29% and 100% (Table S1 in the Supporting Information).

### Guest Encapsulation

Before attempts were made to encapsulate the large chiral agrochemicals **D** and **E**, the encapsulation of model compounds that contain similar chemical fragments, but a smaller structure, was investigated. These encapsulation experiments would allow insight into the effect of the size of the guest molecules on the guest encapsulation time. They would also provide information on the difference in the position the guests preferentially occupy within the host framework’s pores. To this end inclusion complexes were produced through the encapsulation of target compounds **A**–**C** into the pore of the host crystalline sponge **2**.

**Figure 2:**
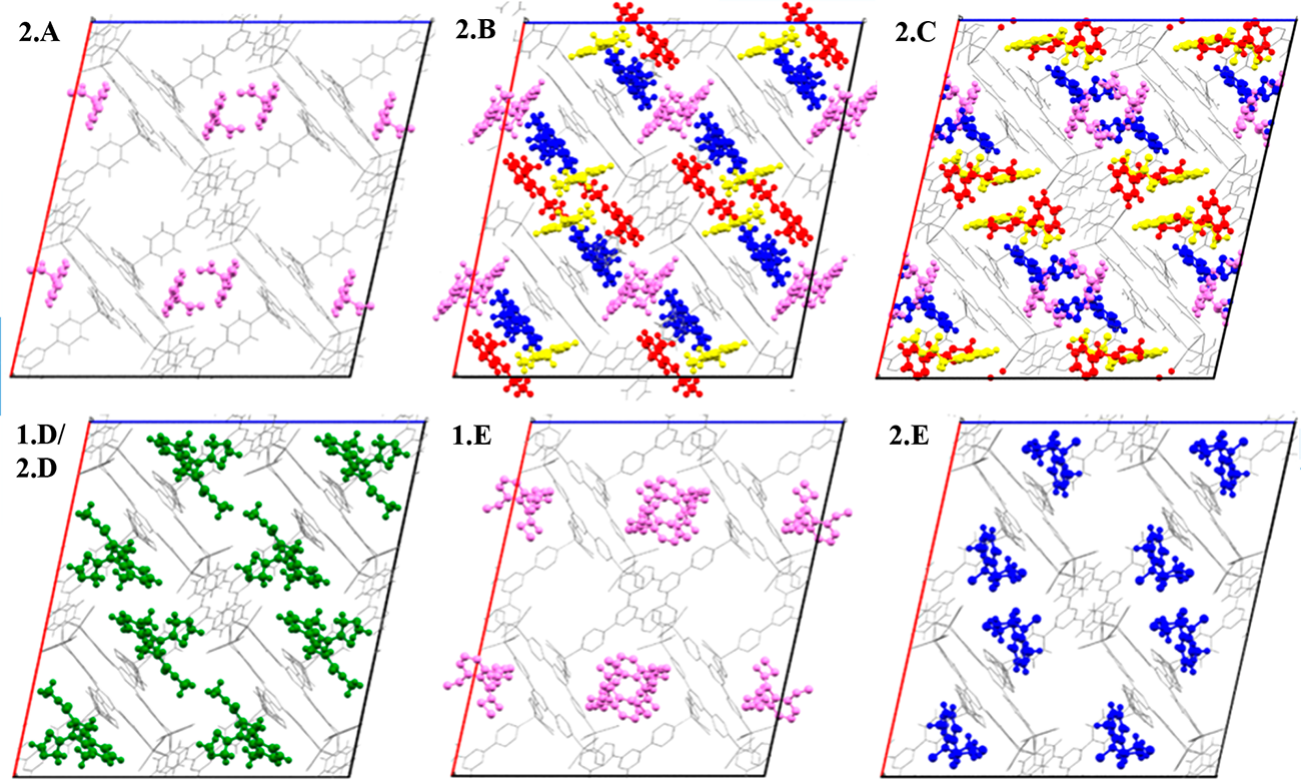
Crystal structure unit cell plots viewed down the crystallographic *b* axis of the inclusion complexes: (**2.A**) phenylacetaldehyde in {[(ZnBr_2_)_3_(TPT)_2_].*x*(CHCl_3_)}*_n_*; (**2.B**) N-ethyl-*o*-toluidine in {[(ZnBr_2_)_3_(TPT)_2_].*x*(CHCl_3_)}*_n_*; (**2.C**) methyl phenylacetate in {[(ZnBr_2_)_3_(TPT)_2_].*x*(CHCl_3_)}*_n_*; (**1.D**/**2.D**) metalaxyl-M in {[(ZnI_2_)_3_(TPT)_2_].*x*(CHCl_3_)}*_n_* or {[(ZnBr_2_)_3_(TPT)_2_].*x*(CHCl_3_)}*_n_*; (**1.E**) S-metolachlor in {[(ZnI_2_)_3_(TPT)_2_].*x*(CHCl_3_)}*_n_*; (**2.E**) S-metolachlor in {[(ZnBr_2_)_3_(TPT)_2_].*x*(CHCl_3_)}*_n_*. Guest molecules are colored by their positional equivalence, and the host frameworks are shown as gray wireframes.

When the inclusion complex **2.A** is studied, it can be seen in Figure 2 that one molecule of **A** was able to be successfully located and refined within the asymmetric unit with a high occupancy of 90%. Empty pores can be seen in the unit cell diagram in Figure 2 as well as when the unit cell space-filling model is studied (Figure S3 in the Supporting Information). This is due to the presence of disordered chloroform solvent molecules or guests that were encapsulated into the pores of **2** but were too heavily disordered to be successfully located and refined.

**Figure 3:**
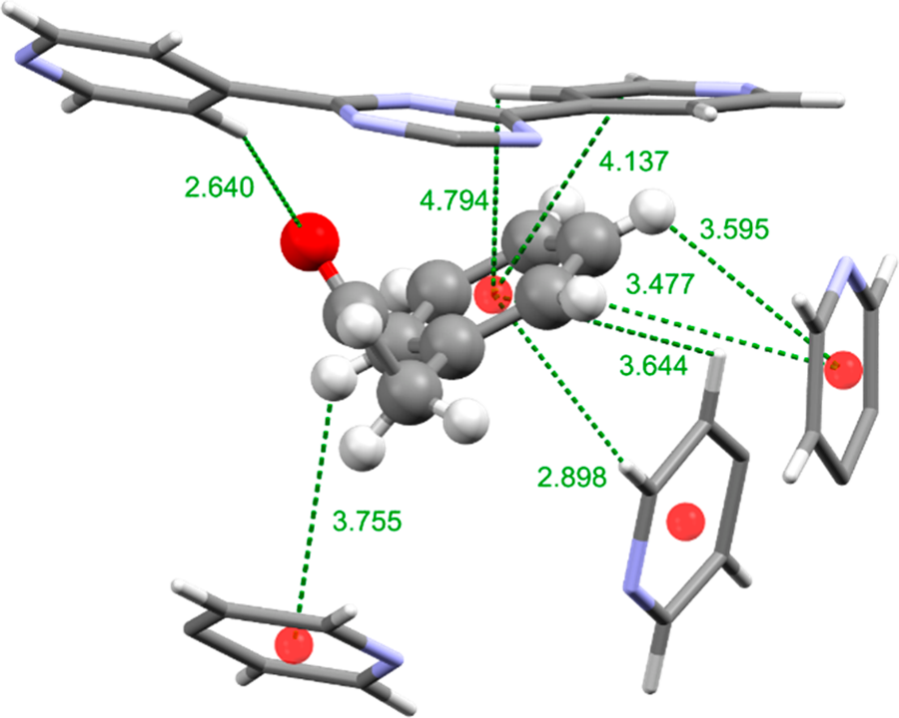
Intermolecular host–guest CH···π and hydrogen-bonding interactions and distances used to order **A** within the pores of **2**. The guest molecule is displayed as a ball and stick model and the host framework as a capped-stick model. Centroids are shown as red spheres and intermolecular interactions as green dotted lines, and interaction distances are shown in angstroms.

An analysis of the intermolecular host–guest interactions (Figure 3) shows that CH···π interactions are the primary forces used in the ordering of **A** within the pores of **2**. In fact, seven CH···π host–guest interactions were formed with four pyridine rings of the TPT linker molecules. It was also observed that the aldehyde oxygen atom occupies a site 2.640 Å from a TPT pyridyl hydrogen atom. This is within the expected range of a hydrogen bond. As carbon is not a very electronegative atom, the hydrogen bond formed would be weak (Figure 3).

The encapsulation of guest **B** into the pores of the host **2** was initially indicated by the crystals changing color from colorless to red shortly after the addition of the guest to the host crystals (Figure S12 in the Supporting Information). This is different from that observed for the other guests encapsulated in this study, where the crystals remained colorless after the guest encapsulation procedure was performed. Color changes have been reported previously in the literature, such as for the encapsulation of guaiazulene by Fujita et al., in which a color change from colorless to blue was observed in the crystals of the host crystalline sponge **1**.^1,2^ Unlike the case for inclusion complex **2.A**, the space-filling unit cell model of **2.B** shows that all available space has been filled. Therefore, it is unlikely that there are heavily disordered guest and/or solvent molecules within this structure.

In comparison to inclusion complex **2.A**, four crystallographically unique molecules of **B** were successfully located and refined within the asymmetric unit of complex **2.B**. Three of the four guest molecules were refined with 100% occupancy, which is slightly higher than that observed for guest **A**; the fourth molecule of **B** was refined with 50% occupancy. When the unit cell diagrams of complexes **2.A** and **2.B** were studied, it was noticed that the violet guest molecules occupy the same pores of the host **2**. When the structures of the host frameworks are superimposed, it can be seen that the acetaldehyde group of **A** and the *N*-ethyl group of **B** are oriented in different directions (Figure S4d in the Supporting Information). The C_Ar_–C bond of the acetaldehyde group of **A** is oriented down the crystallographic *b* axis but also points slightly in the direction of the *a* axis, whereas the *N*-ethyl group of **B** is oriented approximately near parallel to the crystallographic *b* axis (Figure 2 and Figure S4d in the Supporting Information). If hydrogen atoms are added to the model of the violet molecule of **B** (as shown in Figure 5c), it can be clearly seen that **B** was ordered within the host framework by the formation of five CH···π interactions with three TPT pyridyl rings and two guest–guest interactions: one with the red guest molecule and one with the guest molecule displayed in blue (Figure 2 and 5c).

**Figure 4:**
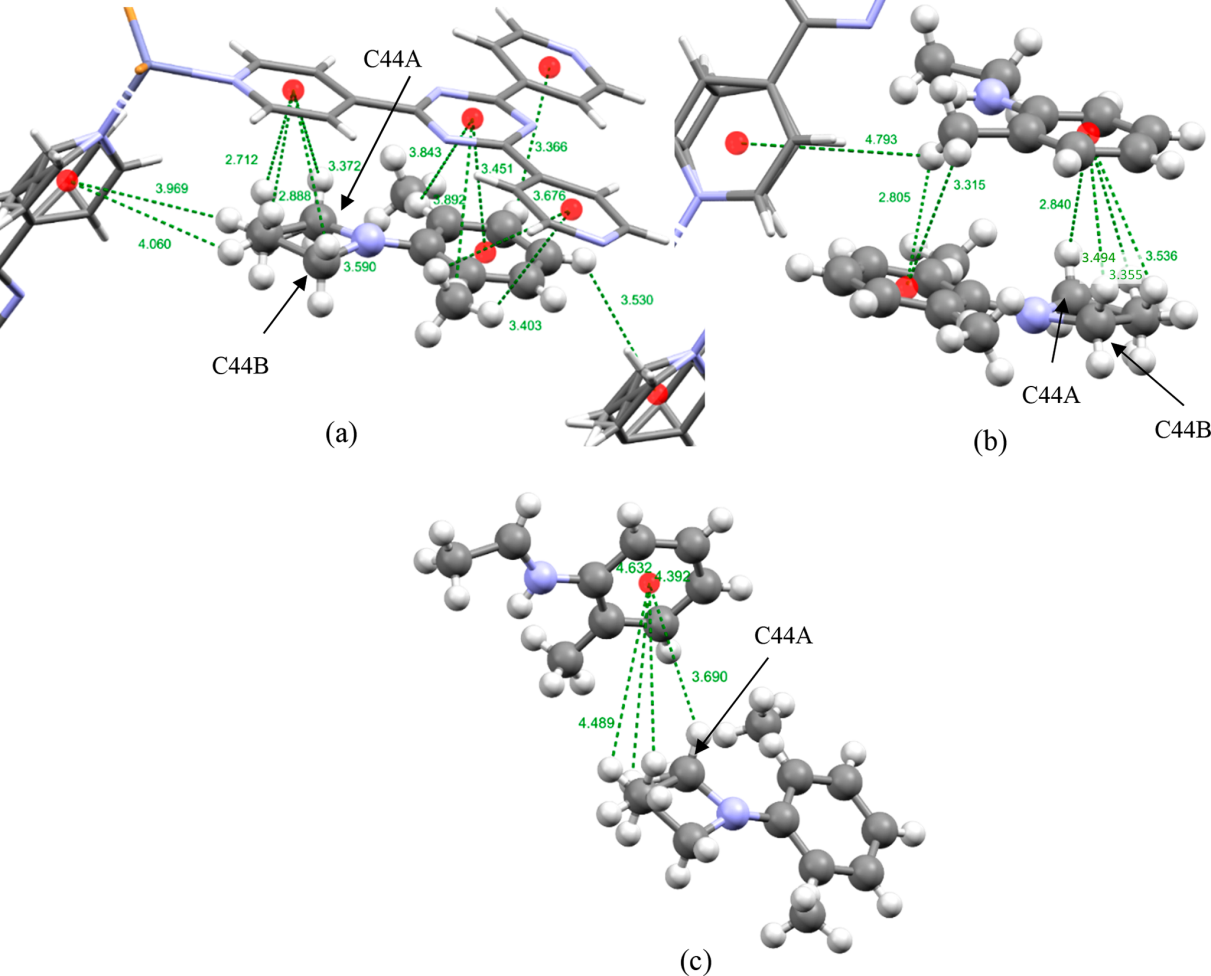
Intermolecular host–guest CH···π and π···π interactions used to order guest **B** displayed in blue in Figure 2 within the pores of **2**. (a) Intermolecular interactions between the blue guest molecule of **B** and the TPT linkers of the host **2**. (b) The guest molecules displayed with blue coloration (bottom) and red coloration (top) in Figure 2. (c) Guest molecules of **B** displayed in blue (bottom) and yellow (top) in Figure 2. The guest molecules are displayed as a ball and stick model and the host framework as a capped-stick model. Centroids are shown as red spheres and intermolecular interactions as green dotted lines, and interaction distances are shown in angstroms.

The guest molecule of **B** displayed in blue in Figure 2 exhibits disorder; specifically the carbon atoms C37 and C44 are disordered over two positions with occupancies of 70% and 30%, which can be observed in Figure 4a. The disordered site with higher occupancy perhaps unsurprisingly shows many more short intermolecular contacts with the framework and other guests than does the site with lower occupancy (Table S2 in the Supporting Information).

**Figure 5:**
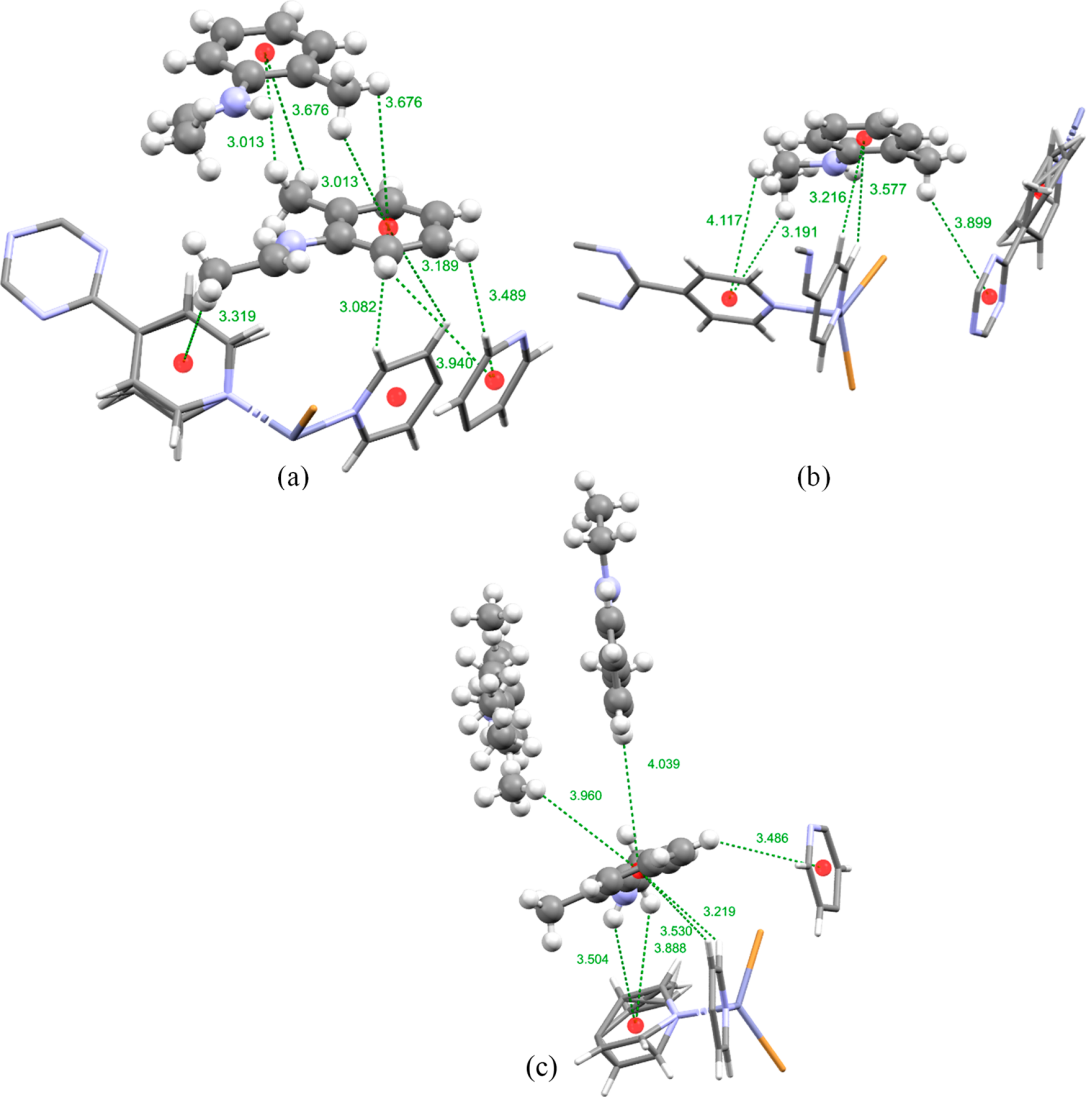
Intermolecular host–guest and guest–guest interactions of (a) **B** in yellow, (b) **B** in red, and (c) **B** in violet, as displayed in Figure 2. The guest molecules are displayed as a ball and stick model and the host framework as a capped-stick model. Centroids are shown as red spheres and intermolecular interactions as green dotted lines, and interaction distances are shown in angstroms.

The other two encapsulated guest molecules do not display any disorder. As was mentioned previously, the guest molecule displayed in yellow in Figure 2 is ordered through a series of CH···π interactions with the guest molecule shown in blue (Figure 2), as presented in Figure 4c. In addition to this the yellow guest molecule forms several CH···π and a π···π intermolecular interactions with three pyridine rings of the TPT linkers and a second yellow guest molecule which is related by inversion symmetry (displayed in Figure 5a). The guest molecule displayed in red was ordered solely by CH···π intermolecular interactions: six guest–guest interactions mentioned previously formed with the blue guest molecule shown in Figure 4b, additionally another CH···π interaction was formed with the violet guest molecule (Figure5c), and a further six host–guest CH···π interactions were formed with three TPT pyridine rings as well as a TPT triazine ring displayed in Figures 4b and 5b.

**Figure 6:**
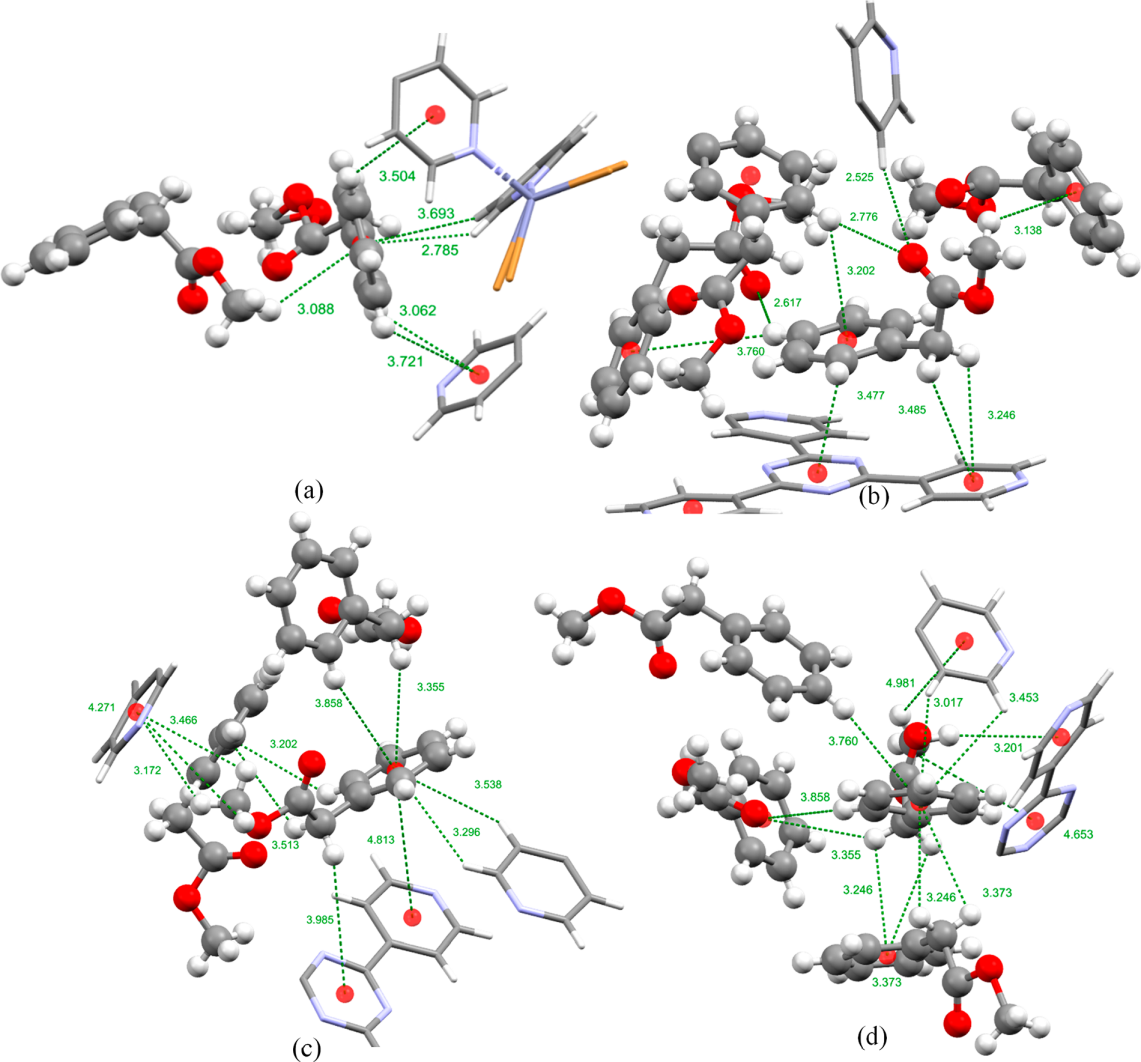
Intermolecular host–guest and guest–guest interactions and distances formed in complex **2.C** guest molecules: (a) violet, (b) blue, (c) red, and (d) yellow as displayed in Figure 2. The host framework is displayed as a capped stick model and the guest molecules as a ball and stick model. Centroids are displayed as red spheres, intermolecular interactions are represented by green dotted lines, intermolecular interactions that could not be fully modeled are represented by a red dotted line, and the distances are shown in angstroms.

When the unit cell diagrams in Figure 2 were studied, four molecules of guest **C** were observed to have been encapsulated into the pores of the host framework **2** with occupancies between 50 and 59%, forming the inclusion complex **2.C.** The positions the guests occupy in the MOF pores show great similarity to those seen in inclusion complexes **2.A** and **2.B**. In fact, each guest position seen in complexes **2.A** and **2.B** is also filled with a molecule of **C** in complex **2.C**, though some small differences in orientation are present (Figures S4–S8 in the Supporting Information). Such examples are the red guest molecules of **B** and **C**. The guest phenyl rings occupy very similar positions (Figure S5 in the Supporting Information), as such the CH···π interactions observed with a pyridyl ring of the TPT linker are comparable with CH···π**_B_** distances of 3.216 and 3.577 Å (Figure 5b) and the CH···π**_C_** distances of 3.296 and 3.538 Å (Figure 6c). On the other hand, the main functional groups occupy different positions; the methyl acetate group of **C** is positioned nearly parallel with the crystallographic *c* axis in comparison to the approximate *ac* direction that the *N*-ethyl group of **B** exhibits (Figure 2). These differences also give rise to different host–guest interactions. Overall, the red molecule of **C** was ordered within the pores of **2** by one π···π and several CH···π interactions with three TPT pyridyl rings, one CH···π interaction with a TPT triazine ring, and guest–guest CH···π interactions with guests displayed in blue and yellow in Figure 2.

The unit cell diagrams in Figure 2 of complexes **2.B** and **2.C** show that the guest molecules displayed in blue are in similar positions but in the case of **C** are slightly offset with respect to the TPT linker in comparison to **B**, which occupies a position directly above the TPT linker. In a closer look it can be seen that the methyl acetate group of **C** points roughly parallel to the crystallographic *c* axis (Figure 2), allowing for the formation of guest–guest hydrogen bonding and CH···π interactions with the yellow, red, and violet and guest molecules, respectively (Figure 6b). The *N*-ethyl group of **B**, on the other hand, was oriented roughly parallel to the crystallographic *b* axis (Figure 2) and occupies a position directly above the TPT linker, facilitating the formation of many intermolecular CH···π interactions as seen in Figure 4a.

The guest molecule of **C** displayed in yellow in Figure 2 occupies a near identical site to their guest **B** counterpart. The yellow guest molecule of **C** was able to be fully refined with an occupancy of 58%. This molecule is ordered within the host framework by the formation of five host–guest CH···π interactions with the TPT linkers of the host framework. In addition seven guest–guest interactions were formed: four with another yellow guest molecule which was related by a 2-fold rotation, two CH···π interactions with the guest molecule displayed in red and one CH···π interaction formed with the molecule displayed in blue coloration (Figure 6.d). When the frameworks of **2.B** and **2.C** are superimposed it can be seen that the phenyl rings of the yellow guest molecules occupy similar positions but the guests functional groups are orientated in different directions (Figure S6, Supporting Information). The N-ethyl group of **B** is orientated in such a way that it is nearly parallel to the crystallographic *b* axis whereas, the methyl acetate group of **C** is orientated in a similar direction to the methyl group of **B** which is nearly parallel to the crystallographic *c* axis (Figures 2 and S6, Supporting Information).

The last guest molecule of **C**, displayed in violet in Figure 2, shows disorder and occupies a site nearly identical with those of their **A** and **B** counterparts. The guest molecule displayed in violet in **2.C** is ordered through a series of host–guest CH···π and π···π interactions with three pyridyl rings of the TPT linkers and a guest–guest CH···π interaction with the blue guest molecule. This guest molecule also shows disorder in the oxygen atom O6 position, which was found in two positions, each with equal occupancies. Superimposing the framework of **2.C** on that of **2.A** reveals that the phenyl rings of the guest molecules in each occupy the same positions in the host pores but that the functional groups are oriented in opposite directions along the crystallographic *b* axis (Figure 2 and Figure S4a in the Supporting Information).

Previous studies by Carmalt et al. highlight the positions guest molecules prefer to occupy in the pores of the host framework **1**.^17,32^ Though the guests discussed so far in this study have been encapsulated into the host framework **2**, it is still appropriate that a comparison be made, as the major difference between the two frameworks is the identity of the halide bonded to the zinc (I and Br for **1** and **2**, respectively); the host framework structures themselves are otherwise nearly identical. When the unit cell diagrams are compared, it can be clearly seen that the guest positions displayed in red, violet, and blue in Figure 2 are similar to the guest positions seen in the unit cell diagrams presented in previous studies.^17,32^ A more in depth analysis shows that the position within the host framework where the guests are displayed in violet (inclusion complexes **2.A** and **2.C**) are also filled with guest molecules in previously reported inclusion complexes with the guests benzene,^17^ naphthalene,^32^ benzyl cyanide,^32^ and benzaldehyde^17^ encapsulated at these sites. The red guest molecule position (as seen in complexes **2.B** and **2.C** in Figure 2) was also observed in inclusion complexes of **1** with encapsulated benzaldehyde,^17^ acetophenone,^32^ benzene,^17^ and benzonitrile.^32^ The blue position is also not unique to the inclusion complexes of this study (**2.B** and **2.C**), as other guests occupying this position also include benzene^17^ and naphthalene.^32^ It must be noted that, while the sites these guest occupy may be very similar, there are differences in the position of the guest functional groups and the degree of disorder experienced. The consistency with which guest molecules occupy these sites confirms the conclusions of previous studies,^17,32^ where it was noticed that guests tend to regularly fill specific positions of the pores of the host framework. This study has shown that at least some of the positions guests occupy when they are encapsulated into the host framework **1** are also common when guest molecules are encapsulated into the host **2**.

### Encapsulation of Metalaxyl-M

Metalaxyl-M is a chiral acylalanine fungicide^26^ commonly used to control diseases in many crops. The commercially sourced compound **D** was not the enantiopure *R* stereoisomer and had a reported enantiomeric purity of ≥90% to <100% purity. **D** was encapsulated into the pores of both host crystalline sponge frameworks **1** (at 50 °C) and **2** (at 25 and 50 °C).^33,34^ Upon encapsulation into both of the host frameworks, the full structure of **D**, with atom connectivity consistent with that published previously,^27,35^ was located and refined with maximum occupancies of 52% in **1.D** and 58% in **2.D**.

**Figure 7:**
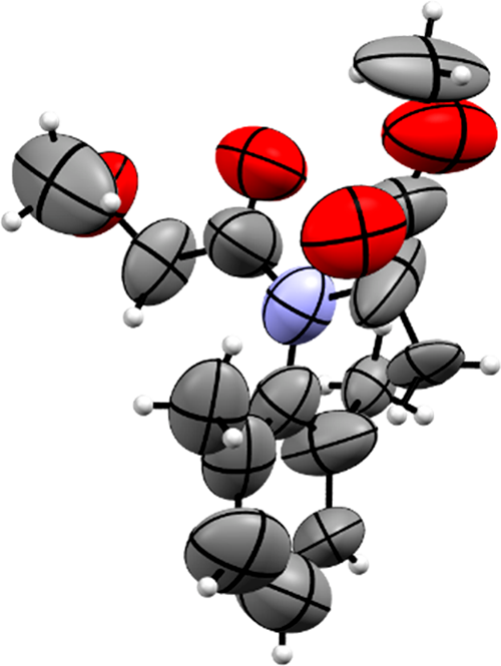
ORTEP diagram with thermal ellipsoids at 50% probability showing the X-ray structure of the encapsulated guest **D**. Carbon atoms are shown in gray, nitrogen atoms are shown blue, and oxygen atoms are shown in red.

**Figure 8:**
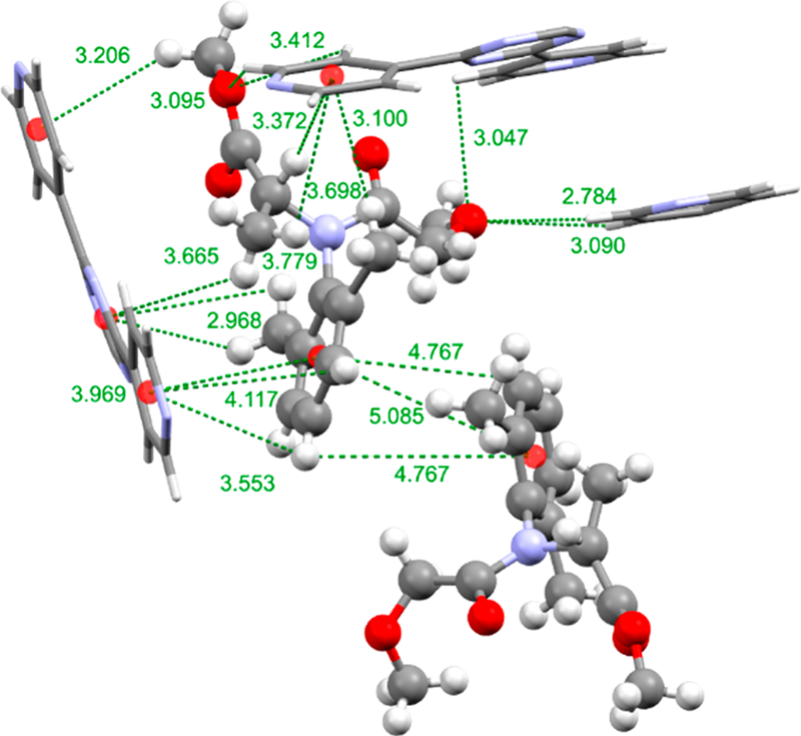
Intermolecular CH···π and π···π interactions formed between the host crystalline sponge framework and guest **D** when **D** was encapsulated into the host framework **2** at 25 °C. The host frameworks are displayed as a capped-stick model and the guest molecules as a ball and stick model. Centroids are shown as red spheres and the host–guest interactions as green dotted lines. The interaction distances are given in angstroms.

One molecule of guest **D** (Figure 7) was able to be located and refined within the asymmetric units for both crystalline sponges **1** and **2**. The molecule of guest **D** displayed in green in Figure 2 was found to occupy the same site of the host pores independent of the host framework and temperature used for guest encapsulation. This is evident when the intermolecular interactions formed are compared between the host framework and guest molecule **D** in Figure S9a–c in the Supporting Information. It can be seen that the intermolecular interactions formed when **D** is encapsulated into both host frameworks, **1** and **2**, are nearly identical with only a slight variation seen in the interaction distances. This was also observed when Figure S9a,b in the Supporting Information were compared, where the guests were encapsulated into the host **2** at 25 and 50 °C, respectively. Guest **D** was ordered within the pores of the hosts **1** and **2** through a large number of CH···π and π···π intermolecular interactions, both between the guest molecule and hosts TPT linker molecules and in guest–guest interactions with another molecule of **D** that is related via inversion symmetry (Figure 8). The position **D** occupies within the host pores is slightly different from that of the blue guest molecules of guests **B** and **C**. Whereas the phenyl ring of the blue molecule of guest **B** occupies a position parallel to the TPT triazine ring (Figure 4a), the phenyl ring of guest **D** can be seen to occupy a space above one of the pyridyl rings at a slight angle, as observed in Figure 8.

### Encapsulation of *S*-Metolachlor

*S*-Metolachlor (**E**) was also encapsulated into both the host crystalline sponges **1** (25 °C) and **2** (50 °C). **E** is a member of the chloroacetamide family of herbicides used to control weeds through mitosis inhibition; during cell division the synthesis of very long chain fatty acids is inhibited to prevent the growth of weed seedling shoots.^25,36^ The commercially sourced compound **E** was not the enantiopure *S* stereoisomer and had a reported enantiomeric purity of ≥85%; also, **E** exhibits a hindered rotation about the C_Ar_–N axis, resulting in atropisomers.^28,35^ To the best of our knowledge, only one other herbicide, Molinate, has had its X-ray structure determined via the CSM; this was performed with a different host MOF, RUM-2.^24^

**Figure 9:**
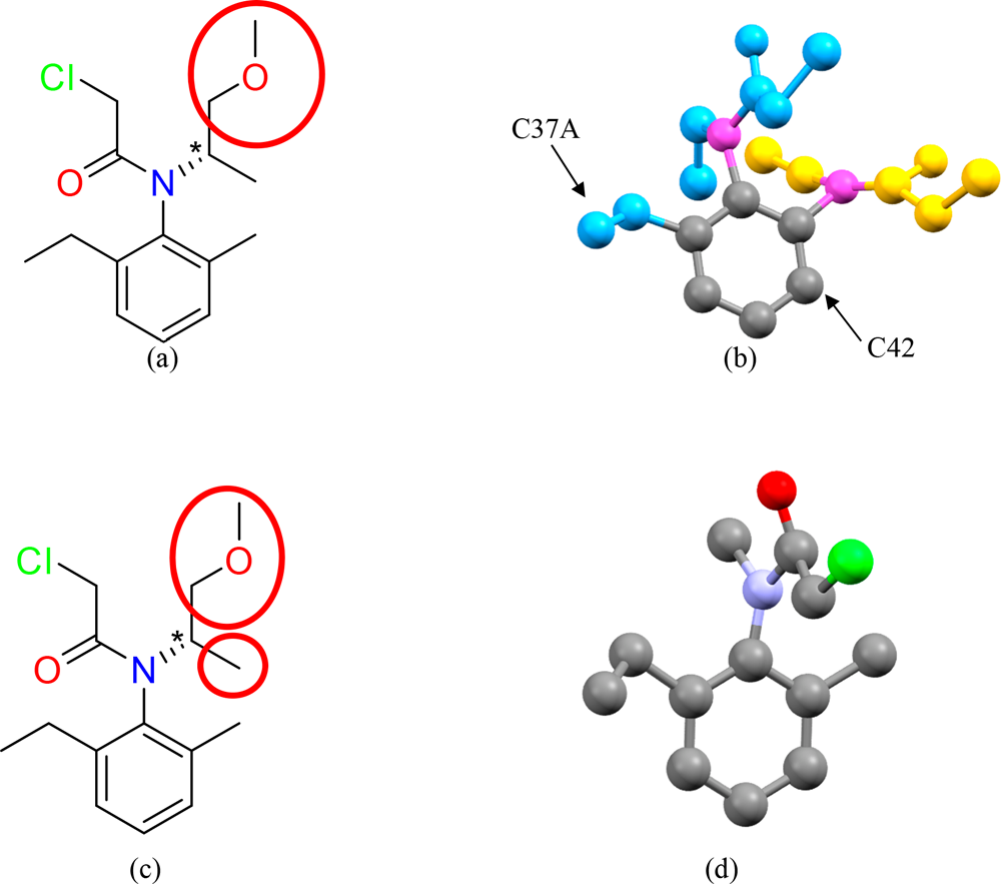
(a) Structure diagram of *S*-Metolachlor with the atoms missing in complex **1.E** shown in a red circle. (b) The disordered crystal structure of **E** when it is encapsulated into **1** at 25 °C. The color of the atoms indicates the disordered components, as shown in blue and yellow. The atoms magenta represent positions that are occupied by both disordered components. (c) Structure diagram of *S*-Metolachlor with the atoms missing in complex **2.E** shown in red circles. (d) Ball and stick model of the crystal structure of **E** when it is encapsulated into **2** at 50 °C.

On encapsulation into the host **1** with an incubation temperature of 25 °C, one molecule of **E** was located in the MOF asymmetric unit. This molecule was refined at 54% occupancy and displayed significant disorder. As shown in Figure 9b, the chloroacetamide group was disordered over two positions, each refined at 27% occupancy (half that of the full guest molecule). The carbon atoms also occupy approximately the same sites as the nitrogen atoms of the chloroacetamide groups (shown in magenta); these carbon atoms correspond to the methyl and ethyl groups and are also disordered. The terminal methyl groups that must be present on the disordered ethyl substituents could not be convincingly located (Figure 9b) in the final electron density map. On encapsulation into the host **2** at 50 °C one molecule of guest **E** was also able to be located within the pores of the crystalline sponge. In this case the guest molecule was refined at a lower occupancy of 33% and no disorder was observed (Figure 9d).

Guest **E** has two different chiral elements: a stereocenter and a chiral axis from a hindered rotation around the C_Ar_–N bond (atropisomerism). This means that there are in fact four different stereoisomers possible for metolachlor (a*R*,1′*S*, a*S*,1′*S*, a*R*,1′*R*, and a*S*,1′*R*).^28,35,37^ In both of the complexes **1.E** and **2.E** an a*R* and an a*S* atropisomer can be observed. All of the atoms attached to the stereocenters (indicated with an asterisk in Figure 9a,c) could not be located within the X-ray structures (Figure 9b,d); therefore, it was not possible to determine the chiral symmetry at this stereocenter for both atropisomers. While it would be exciting to observe the presence of particular atropisomers in the structures of **1.E** and **2.E** due to the disorder present in each, it is impossible to conclude that there has been preferential absorption of specific atropisomers and the likelihood is that all four are present in the crystals.

**Figure 10:**
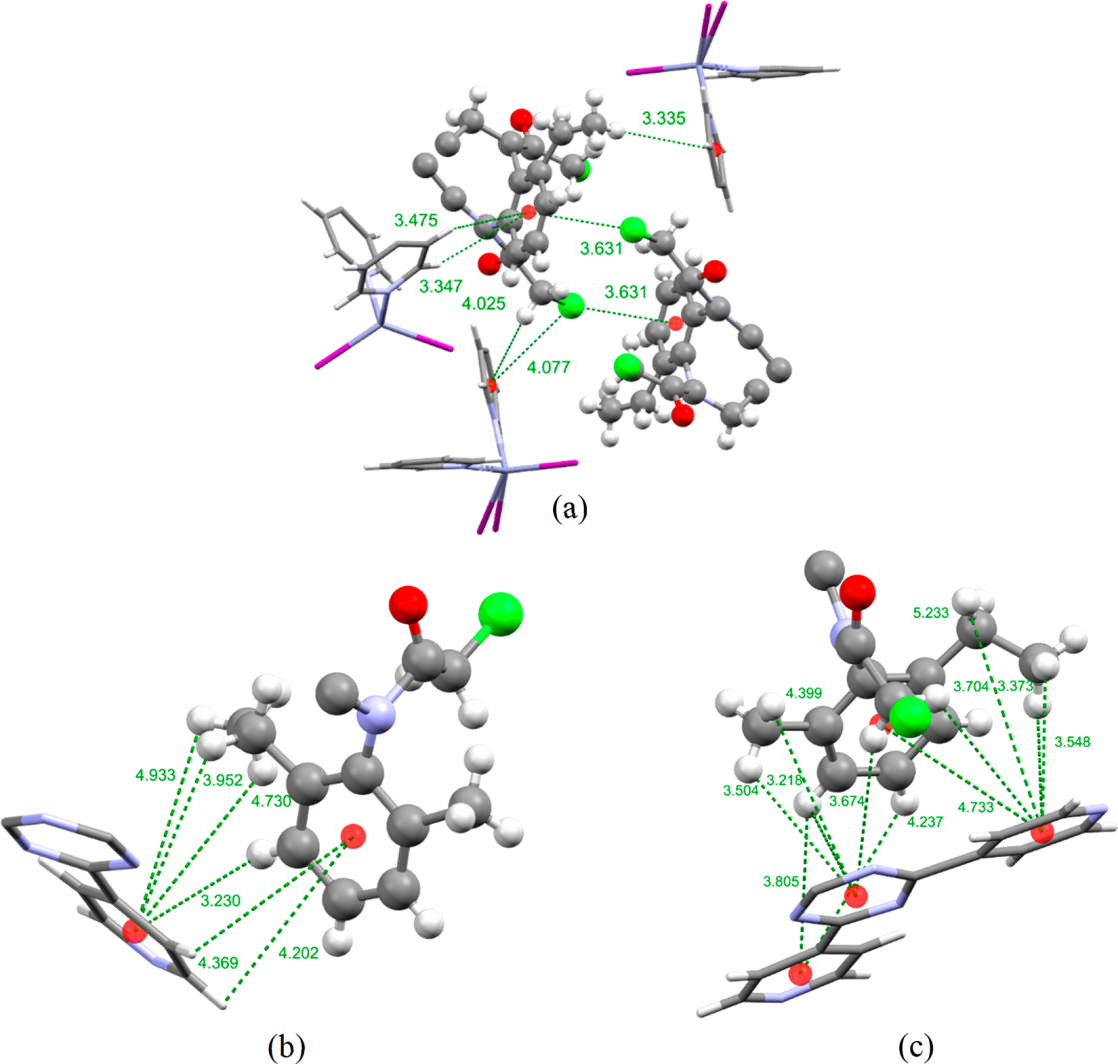
Intermolecular CH···π and π···π interactions formed between the guest molecule and the host framework in inclusion complexes: (a) **1.E**; (b, c) **2.E**. The host framework is displayed as a capped-stick model, and the guest molecules are shown as ball and stick models. Centroids are shown as red spheres. The intermolecular interactions are displayed as green dotted lines, and the distances are given in angstroms.

The position that **E** occupies within the MOF pores varies in complexes **1.E** and **2.E**. In complex **1.E** the guest **E** occupies a position similar to that seen in complexes **2.A**, **2.B**, and **2.C** displayed in violet in Figure 2. The guests **A**, **B**, and **C** are oriented differently from that of guest **E**, as can be observed on a close inspection of the unit cell diagrams in Figure 2 and Figures S4b,c,f in the Supporting Information. Both of the disordered parts of the chloroacetamide group of **E** overlap with the position the phenyl rings occupy in inclusion complexes **2.A** and **2.C**; for **2.B** one of the chloroacetamide groups overlaps with the phenyl ring of **B**. In inclusion complex **1.E**, the guest is ordered within the pores through a series of host–guest CH···π intermolecular interactions with two pyridine rings of the host framework TPT linker. Additionally, two Cl···π interactions can be observed with a TPT pyridine and a second guest molecule, which is related to the first by inversion symmetry (Figure 10a).

In complex **2.E** the guest occupies a site similar to that of the blue guest molecule of **B** (complex **2.B**), though there is a slight difference in orientation. Whereas guest **B** occupies a position above the TPT triazine ring so that the planes of the phenyl and triazine rings are parallel, guest **E** occupies a position above the triazine ring of the TPT linker but, as is illustrated in Figure 10b,c and Figure S7b in the Supporting Information, the guest molecule is angled in such a way that the planes of the phenyl ring and triazine ring are not parallel. The difference is due to the presence of the chloroacetamide group, which is almost perpendicular (C39–C44–N13–C46 torsion angle of 91.74°) to the plane of the phenyl ring. This is not the case for guest **B**, where the *N*-ethyl group sits in the same plane as the guest’s phenyl ring. **E** also shares a position similar to that of guest **C** (complex **2.C**). On comparison of the positions shown in Figure S7c it can be seen that the guests occupy a position next to each other over the same triazine ring of TPT. **C** is also parallel to the plane of the TPT linker, in contrast to the slight angle observed with **E**. The difference in the observed position guest **E** occupies and the difference in disorder observed within inclusion complexes **1.E** and **2.E** is in contrast to the case for guest **D**, where no difference was observed when host **1** or **2** was used for guest inclusion.

### Chiral Guests and Maintaining the Host Framework Inversion Symmetry

All of the inclusion complexes reported in this investigation crystallized with similar unit cell parameters and the same space group symmetry (*C*2/*c*) as seen in both of the as-synthesized host frameworks. Two of the guest compounds (**D** and **E**) encapsulated into these frameworks are chiral; hence, it would be expected that when these compounds are encapsulated into the host framework the inversion symmetry would be lost; this would therefore reduce the space group symmetry of these inclusion complexes from the centrosymmetric space group *C*2/*c* to the noncentrosymmetric space group *C*2. This was not the case; inclusion complexes **1.D**, **2.D**, **1.E**, and **2.E** were all found to crystallize in the space group *C*2/*c*.

The commercially sourced compounds **D** and **E** were not the enantiopure *R* and *S* stereoisomers, respectively, and had reported enantiomeric purities of 85+%. Additionally **E** exhibits a hindered rotation about the C_Ar_–N axis, resulting in atropisomers.^28,35^ The results of this investigation show that it is more favorable for the host to encapsulate equal quantities of both enantiomers into the host framework than it is to break the inversion symmetry, even when one enantiomer is only present in small quantities.

Similar results have been previously reported by de Gelder et al. for the encapsulation of 90% pure (+)-camphene into the host framework **1**.^38^ The host’s C2/*c* symmetry was retained with four molecules of (+)-camphene and four molecules of (−)-camphene reported within the unit cell; this is comparable to that observed for guests **D** and **E**.^38^ Another example is the encapsulation of 99% pure (−)-carvone into the host framework RUM-2.^31^ Despite the very high enantiopurity of the guest, the host–guest complex was observed to crystallize in the space group *C*2/*c*.

### Effect of Temperature

Initially encapsulation experiments for the inclusion of guests **D** and **E** were performed at 25 °C. At this temperature, even after 45 days, crystal structures were produced where only the solvent chloroform was able to be located and refined within the pores of the host framework (Table S1, in the Supporting Information). For the encapsulation of guest **D**, 90 days of incubating the host crystals in neat liquid **D** was required for a sufficient quantity of the guest to enter the host’s pores, allowing for the successful location and refinement of **D** following SCXRD analysis (Table 1). A similar observation was made when **E** was encapsulated at 25 °C ,where 53 days of crystal incubation was required (Table 1). However, a complete structure of **E** was not able to be elucidated even after 142 days of soaking (Table S1 in the Supporting Information). These are exceedingly long encapsulation times; therefore, the use of a higher temperature for guest encapsulation was investigated.

**Table 1:** Encapsulation Time Required to Generate Sufficient Guest Occupancy to Allow Location and Refinement of the Guest Molecule(s)

host framework	guest	incubation time at 25 °C/days	incubation time at *50 °C/days*
**2**	**A**	N/A	14
**2**	**B**	N/A	12
**2**	**C**	N/A	17
**1**	**D**	N/A	21
**2**	**D**	90	19
**1**	**E**	53	crystals degraded after 21 days
**2**	**E**	N/A	16

The hypothesis was that using higher temperatures would improve the diffusion kinetics and decrease guest encapsulation times. To this end, guest encapsulation experiments were repeated at 50 °C. A reduction in the time required for guest encapsulation was observed. The encapsulation time required to obtain sufficient **D** inclusion to obtain a complete structure was reduced from 90 days to 21 days for encapsulation into **1** and 19 days for encapsulation into **2** (Table 1). A similar reduction in the time required was seen for the encapsulation of **E**. The time required for encapsulation was reduced from 53 days for encapsulation into **1** at 25 °C to 16 days for encapsulation into **2** at 50 °C (Table 1).

The time required for the encapsulation of guests **D** and **E** at 25 °C was exceptionally long. Encapsulation times ranging from 53 to 90 days would not be acceptable for use in a routine analytical technique used in an academic or industrial setting, such as in the agrochemical research industry. The time required for guest inclusion in the CSM is typically a great deal shorter than those reported here for the encapsulation of guests **D** and **E**; these range from a day to a few weeks.^17,19,32,39−41^ Increasing the temperature at which the encapsulation was performed to 50 °C vastly reduced the time required for the encapsulation of **D** and **E** to times that were closer to those observed in other guest encapsulations reported in the literature.^17,19,32,39−41^

**Figure 11:**
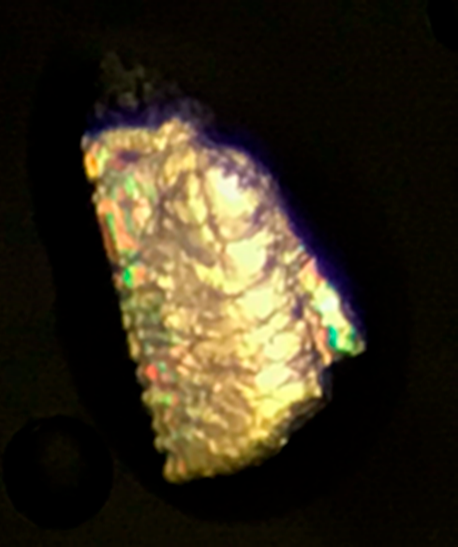
A crystal of the host framework **1** after incubation at 50 °C in the presence of **D** for 21 days.The crystal is shown under polarized light.

Increasing the temperature does come with a caveat, as an increased rate of crystal degradation was observed when framework **1** was used to encapsulate both guests **D** and **E** at 50 °C. A visual inspection of crystals of **1** soaked in **D** exhibited very noticeable signs of crystal degradation at higher temperature. As displayed in Figure 11, the crystal of **1** appears to be physically damaged. SCXRD analysis of the crystals of host **1** after soaking in guests **D** and **E** at 50 °C revealed that both crystals failed to diffract X-rays to high angles (0.84 Å). While a crystal of complex **1.D** was eventually located that could diffract to a high angle, this was not possible for crystals of **1** soaked in neat **E**, where an SCXRD analysis revealed maximum diffraction to a resolution of ∼1 Å. These results are in stark contrast to those when the encapsulation experiments were performed at 25 °C, where even after 142 days of incubation a crystal of complex **1.E** with sufficient quality for SCXRD analysis was able to be identified (Table S1 in the Supporting Information). Encapsulating guests **D** and **E** using the more robust host framework **2**(6,19) with an incubation temperature of 50 °C did not result in similar problems (Table 1 and Table S1 in the Supporting Information).

### Reliability of the Structure Determinations of **D** and **E**

The encapsulation of guests **D** and **E** highlights some of the limitations of the CSM. As was mentioned above, during guest encapsulation both of these compounds damaged the host crystals, creating visually noticeable signs of crystal degradation similar to, but to a lesser extent, that shown in Figure 11. This was observed with both crystalline sponges **1** and **2**, but the host framework **2** was shown to be far more robust, displaying significantly less damage after encapsulation of both **D** and **E**. In many of the crystals (especially **1.E**) this damage would lower the resolution of the diffraction pattern produced to >0.84 Å. All diffraction patterns produced for the reported crystal structures achieved a diffraction resolution of better than 0.84 Å, but the diffraction peaks at high angle were weaker than those shown by an undamaged crystal, such as those obtained for inclusion complexes **2.A**, **2.B**, and **2.C**. As a result the quality of the crystal data collected was lower for guests **D** and **E** than for **A**, **B**, and **C**. This shows that the crystalline sponges **1** and **2** are not fully stable when they are in the presence of the agrochemical compounds **D** and **E**.

A full structure of **D** was able to be refined with maximum guest occupancies of 52% for complex **1.D** and 58% for **2.D** (Table S1). These occupancies thus led to the observation of low-intensity electron density peaks corresponding to the acylalanine group. This increased the uncertainty of the guest’s atomic positions; therefore, during structure refinement a number of bond length restraints (e.g., DFIX) were required for the production of a stable refinement.

The full structure of **E** was not able to be fully located and refined within the inclusion complexes **1.E** and **2.E**; this highlights some of the limitations of the CSM. The low occupancy of the guest molecule in **1.E** and **2.E** led to problems in refining disordered models. In **1.E** the substituents are disordered over two partially overlapping sites. While the data allow the identification of the Cl atom positions in the disordered models, the positions of the light atoms associated with the disorder is problematic. Indeed, our best models have light atoms “missing”. Similar problems have been reported previously.^22,42,43^

Despite the success in fully refining the structure of **D** and locating and refining a partial structure of **E**, the quality of the data obtained and the low occupancy of the guest molecules resulted in a large number of crystallographic restraints being utilized. As a consequence these experiments would not be sufficient for the unambiguous structure determination of an unknown or novel compound. This highlights the current limitations of the CSM, for which, despite the advancements of this technique since it was first reported in 2013,^1,2,19,20^ more research still needs to be done in order to develop the CSM into a routine analytical method for the crystal structure determination of noncrystalline compounds.

The encapsulation of **E** also highlights the need to diversify the crystalline sponge method with a range of host frameworks that are stable in the presence of a variety of compounds with different chemical properties (e.g., hydrophobic, hydrophilic, and nucleophilic); this would aid in removing the limitation of host framework instability. Additionally, exploring the use of frameworks capable of forming stronger interactions (e.g., coordination bonds) with a guest molecule may aid in guest ordering and reduce the chance of guest molecules being disordered. This should improve the data quality, leading to an unambiguous identification of guest molecule stereochemistry.

## Conclusion

The work presented here highlights both the possibilities and the current limitations of the crystalline sponge method. The X-ray structures of four liquid guest molecules, including the agrochemical fungicide active ingredient Metalaxyl-M, have been successfully determined by encapsulation into the pores of the host frameworks {[(ZnX_2_)_3_(2,4,6-tri(4-pyridyl)-1,3,5-trazine)_2_].*x*(solvent)}*_n_* (X = I, Br). The encapsulation of Metalaxyl-M, which to the best of our knowledge is the first fungicide active ingredient to have its X-ray structure determined via the CSM, further establishes the potential of the CSM for the characterization of noncrystalline or hard to crystallize compounds in agrochemical research and product development. In particular the CSM can be seen as a possible option for the structural elucidation of the metabolites of crop protection active ingredients.

The guest molecules in this study were shown to occupy similar sites within the pores of the host frameworks, displaying only small positional and rotational differences, with the exception of guest **D**. These sites are also very similar to those observed in previous studies performed by Carmalt et al. on the encapsulation of simple aromatic compounds into the pores of the host **1**.^17,32^ The full structures of the guest molecules **A**–**D** were successfully elucidated with little or no disorder.

It was not possible to locate the full structure of **E** during this study. **E** also displayed significant disorder upon encapsulation into **1** at 25 °C, which significantly increased the difficulty of structure refinement. The refined molecules of guests **D** and the partial molecules of **E** both conform to structures published previously.^27−29,35^ Encapsulation of the chiral guests **D** and **E** did not cause a loss of the host framework inversion symmetry as would be expected; instead, it was shown to be more favorable for both enantiomers to be encapsulated. Performing the encapsulation of guests **D** and **E** at 25 °C took an exceptionally long time that would not be acceptable for a routine analytical procedure. Increasing the encapsulation temperature to 50 °C managed to significantly reduce the time required to a much more acceptable time period; however, this also caused increased crystal quality deterioration. The more robust nature of **2** was demonstrated through the successful encapsulation of guests **A**–**D** at 50 °C, whereas the framework of **1** was much less stable; therefore, the crystal quality of **1** decreased significantly when it was soaked in **E** at 50 °C. Due to the difficulties encountered during the structure refinement of **E**, more research into improving the crystalline sponge method needs to be performed before this technique can be confidently applied to the unambiguous structure determination of unknown agrochemical active ingredient compounds.

## Experimental Procedures

### Host MOF Synthesis and General Procedure for Guest Inclusion

The synthesis of **1** was performed following a procedure from the literature.^30^ The crystalline sponge **2** was synthesized using a method adapted from the literature.^19,30^

A 13 × 100 mm borosilicate test tube was charged with chloroform (4.2 mL) and 2,4,6-tris(4-pyridyl)-1,3,5-triazine (6.3 mg, 0.02 mmol). This mixture was placed into a sonication bath for 10 min to allow the 2,4,6-tris(4-pyridyl)-1,3,5-triazine to dissolve. After sonication the mixture was filtered through a glass pasture pipet plugged with cotton wool into another test tube. A 0.5 mL layer of MeOH was then carefully layered on top using a 1 mL glass syringe creating a clear interface between the two layers. Then 1 mL of a 0.03 M of ZnBr_2_ in MeOH solution was carefully layered on top of the neat MeOH by use of a glass syringe, creating another clear interface between the layers. Dura-seal film was used to cover the test tube before placing it into an incubator at 5 °C for 7 days. A glass Pasteur pipet was then used to gently nudge the crystals off the side of the test tube the crystals were then placed into a 14 mL screw-capped vial with 10 mL of chloroform. The sealed vial of crystals was stored in a 25 °C incubator for a minimum of 7 days before use in guest encapsulation experiments.

A small number of crystals was placed into a 14 mL screw-capped vial. The chloroform storage solvent was carefully removed using a glass pipet immediately followed by the addition of 1 mL of neat guest into the vial, submerging the MOF crystals. The vial was then sealed and placed in an incubator at a specific temperature and time (Table S1 in the Supporting Information) before good-quality block or rod-shaped crystals were chosen for SCXRD analysis.

### Crystallographic Method

Crystals were pipetted from the guest solution onto a glass microscope slide. Fomblin oil was used to coat the crystals to prevent them from drying out while a crystal of appropriate quality for single-crystal X-ray diffraction analysis was selected. The selected crystal was mounted onto a Nylon loop and transferred to the instrument, where it was held in a cryojet stream. An Agilent Super Nova Dual Diffractometer (Agilent Technologies Inc., Santa Clara CA) equipped with Cu Kα radiation (λ = 1.5418 Å) was used to perform the X-ray diffraction analysis at 150 K. The program CrysAlisPro^44^ was used to perform unit cell determinations, absorption corrections, and data reduction. Structures were solved within the OLEX2 GUI^45^ using direct methods in the program SHELXS^46^ and refined using SHELXL^47^ by full matrix least-squares on the basis of *F*^2^. Individual inclusion structure refinement details including the modeling of disorder and the assignment of guest occupancies can be found in the Supporting Information.

## Supporting Information Available

Crystal data and refinement details, space-filling unit cell diagrams, guest position comparison diagrams, encapsulation conditions, and experimental considerations (PDF)

## Accession Codes

CCDC 2046167–2046172 and 2046400 contain the supplementary crystallographic data for this paper. These data can be obtained free of charge via www.ccdc.cam.ac.uk/data_request/cif, or by emailing data_request@ccdc.cam.ac.uk, or by contacting The Cambridge Crystallographic Data Centre, 12 Union Road, Cambridge CB2 1EZ, UK; fax: +44 1223 336033.
