# Supplementary material for: Applying the Crystalline Sponge Method to Agrochemicals: Obtaining X-ray Structures of the Fungicide Metalaxyl-M and Herbicide S-Metolachlor
Source: Cryst Growth Des. 2021 Apr 13;21(5):3024–36. doi: 10.1021/acs.cgd.1c00196 (PMC8154245; doi:10.1021/acs.cgd.1c00196)
Supplement: Supplementary file 1 — cg1c00196_si_001.pdf [file cg1c00196_si_001.pdf]

Supporting Information

Applying the Crystalline Sponge Method to Agrochemicals: Obtaining X-ray Structures of the Fungicide Metalaxyl-M and Herbicide S-Metolachlor

Richard D. J. Lunn,[†] Derek A. Tocher,[†] Philip J. Sidebottom,[‡] Mark G. Montgomery,[‡] Adam C. Keates,[‡] Claire J. Carmalt[*†]

[†]UCL Department of Chemistry, 20 Gordon Street, London, WC1H 0AJ
[‡]Syngenta, Jealott's Hill International Research Centre, Bracknell, Berkshire, RG42 6EY

Corresponding Author Address: UCL Department of Chemistry, 20 Gordon Street, London, WC1H 0AJ. Fax: +44 (0)20 7679 7463. Tel: +44 (0)20 7679 7528

Email: c.j.carmalt@ucl.ac.uk

Contents

	Page
**S1. Experimental Considerations**	**2**
**S2. Guest Encapsulation Experiments**	**4**
**S3. Blue Guest B Disordered Components Intermolecular Interactions**	**5**
**S4. Space-fill Unit Cell Diagrams**	**6**
**S5. Guest Position Comparison**	**7**
**S6. Metalaxyl-M Inclusion Complexes Intermolecular Interactions**	**11**
**S7. General Crystal Structure Refinement Details**	**12**
**S8. Crystallographic Tables and Individual structure refinement details**	**13**
**S9. References**	**28**

S1. Experimental Considerations

To obtain the best quality crystal diffraction data it is imperative to maintain the highest possible quality of the host MOF crystals. To this end all the guest encapsulation experiments were performed in temperature controlled incubators. Controlling the temperature is important to ensure the temperature fluctuations that occur naturally during the day do not potentially create cracks and damage the MOF crystals. The crystalline quality of the MOFs was monitored through visual inspection of the crystal through a microscope and through examination of the diffraction frames produced during SCXRD analysis. Visual inspection of the crystals was performed to look for any obvious signs of crystal degradation, for example cracks on the surface of the crystals such as those seen in Figure S1. After the visual inspection of the crystals was completed, they were subjected to preliminary SCXRD examination and the diffraction pattern assessed. Good quality diffraction peaks should be observed, these peaks should be sharp and diffract to a resolution at least 0.84 Å (Figure S2). During the preliminary diffraction scan and the pre-experiment that follows the crystals unit cell parameters are calculated from the diffraction pattern collected. For a good quality crystal, the unit cell parameters should show a high percentage match with the obtained diffraction pattern.

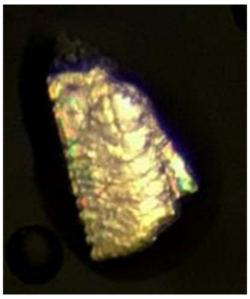

Figure S1. A damaged crystal shown under polarised light of the host framework **1** after incubation in **D** at 50 °C for 21 days.

During guest encapsulation the potential inclusion complexes were analysed via SCXRD to assess whether the guest encapsulation had been successful. For each target compound the amount of time required for encapsulation into the host framework is different. Therefore, crystals from each guest encapsulation experiment had to be analysed on several occasions with increasing encapsulation times. This allowed more time for a sufficient quantity of the target compound to enter the hosts pores increasing the possibility for the guest to be located within the hosts framework (Table S1).

Chloroform was used as the host frameworks pore solvent due to it being present in the pores of the as-synthesised MOF and is compatible with all of the target compounds in this investigation. Chloroform is also a labile solvent as it is only possible for a small number of weak CH···π intermolecular interactions to be formed with the host framework. This helps the process of guest encapsulation by allowing the chloroform solvent to leave the hosts pores creating room for the guest molecules to enter. The guest molecules should then out compete the solvent molecules by forming stronger and more numerous intermolecular interactions (CH···π and π···π) with the host framework. The lability of the chloroform solvent also reduces the chance of a guest molecule and a solvent molecule occupying the same average position. If this occurs it greatly increases the challenge of

solving the crystal structures. Neat liquid guests were also used during guest encapsulation as this produces the highest possible concentration gradient for the guest to enter the MOF.

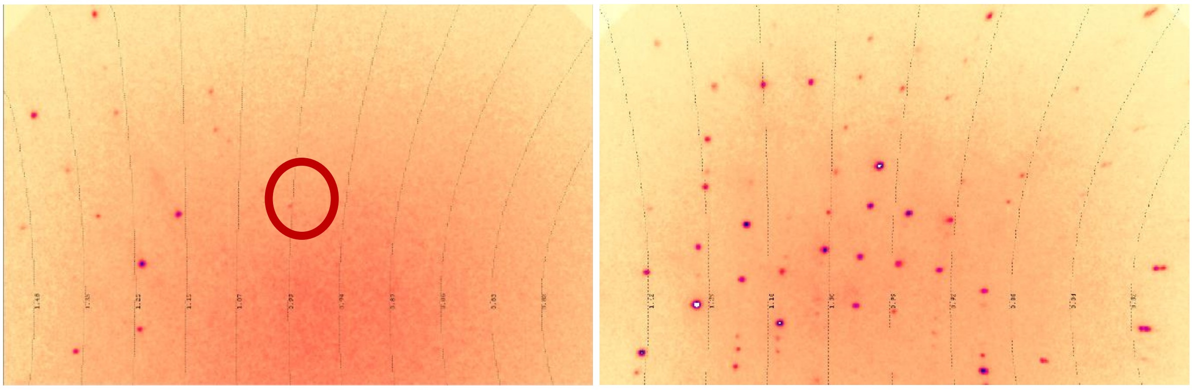

(a) (b)

Figure S2. (a) Diffraction pattern of a damaged crystal where the crystal could not diffract X-rays beyond approx. 1 Å. (b) A good quality diffraction pattern where the crystal can diffract X-ray beyond 0.84 Å (diffraction pattern from complex **2.D**).

S2. Guest Encapsulation Experiments

Table S1. Conditions used for the encapsulation of the guest molecules into the host MOFs and the results produced.

Inclusion Complex	Guest Molecule	Incubation Temperature / °C	Incubation Time / days	Number of Guest Molecules (asymmetric unit)	Guest Occupancy / %	Notes	CCDC number
**2.A**	Phenylacetaldehyde	50	14	1	90		2046167
**2.B**	N-ethyl-*o*-toluidine	50	12	4	100, 100, 100,50	Crystal colour change from colourless to red	2046400
**2.C**	Methyl phenylacetate	50	17	4	50, 52, 58, 59		2046168
**2.D**	Metalaxyl-M	25	45	0	0	Guest not located	
**2.D**	Metalaxyl-M	25	60	1 incomplete (missing one carbon atom)	47		
**2.D**	Metalaxyl-M	25	90	1	55		
**2.D**	Metalaxyl-M	25	135	1	52		
**1.D**	Metalaxyl-M	50	21	1	52		2046169
**2.D**	Metalaxyl-M	50	19	1	43		
**2.D**	Metalaxyl-M	50	35	1	58		2046172
**1.E**	S-Metolachlor	25	53	1 incomplete	50		
**1.E**	S-Metolachlor	25	112	1 incomplete	49		
**1.E**	S-Metolachlor	25	142	1 incomplete	54		2046170
**1.E**	S-Metolachlor	50	21	N/A	N/A	Crystal Deteriorated	
**2.E**	S-Metolachlor	50	16	1 incomplete	29		
**2.E**	S-Metolachlor	50	30	1 incomplete	33		2046171
**2.E**	S-Metolachlor	50	37	1 incomplete	33		

S3. Blue Guest B Disordered Components Intermolecular Interactions

Table S2. Intermolecular host-guest and guest-guest interactions exhibited by the disordered atoms of the blue molecule of guest **B**.

Disordered Atom	Intermolecular interactions /Å		
	CH···$\pi_{pyridine}$	CH···π_{yellow}	CH···π_{red}
C37A	3.403, 3.676		
C37B	-	-	-
C44A	3.372	3.690	2.840
C44B	3.590		3.494

S4. Space-fill Unit Cell Diagrams

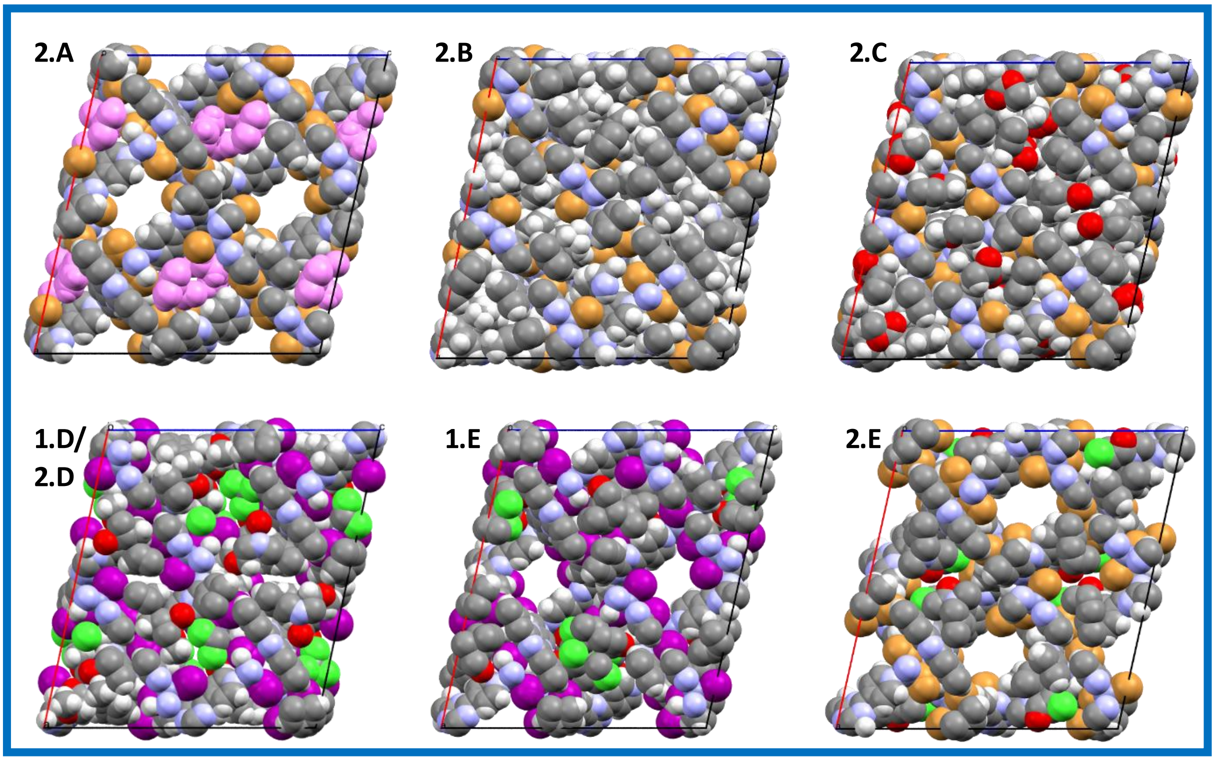

Figure S3. Space-fill unit cell diagrams of the inclusion complex crystal structures viewed down the crystallographic *b* axis.

S5. Guest Position Comparison

The colours of the guest positions can be viewed in Figure 2.

Violet guest molecules

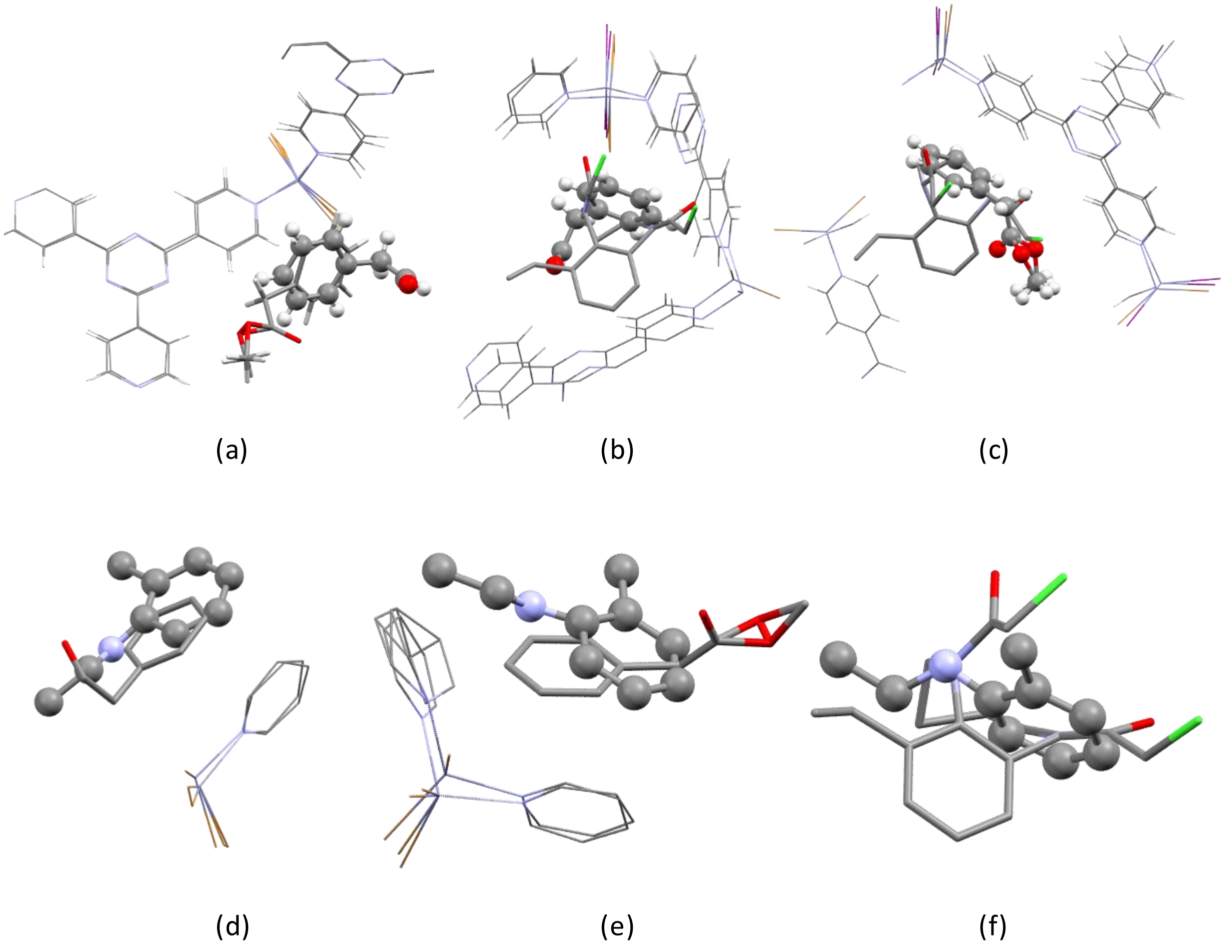

Figure S4. Guest position comparison of the violet guest molecules in complexes: (a) **2.A** and **2.C** where **A** is displayed as a ball and stick model and **C** is displayed as a capped stick model, (b) **2.A** and **1.E** where **A** is displayed as a ball and stick model and **E** is displayed as a capped stick model and (c) **2.C** and **1.E** where **C** is displayed as a ball and stick model and **E** is displayed as a capped stick model, (d) **2.A** and **2.B** where **A** is displayed as a capped stick model and **B** is displayed as a ball and stick model, (e) **2.B** and **2.C** where **B** is displayed as a ball and stick model and **C** is displayed as a capped stick model, (f) **2.B** and **1.E** where **B** is displayed as a ball and stick model and **E** is displayed as a capped stick model. The host frameworks are displayed as a wireframe.

Red guest molecules

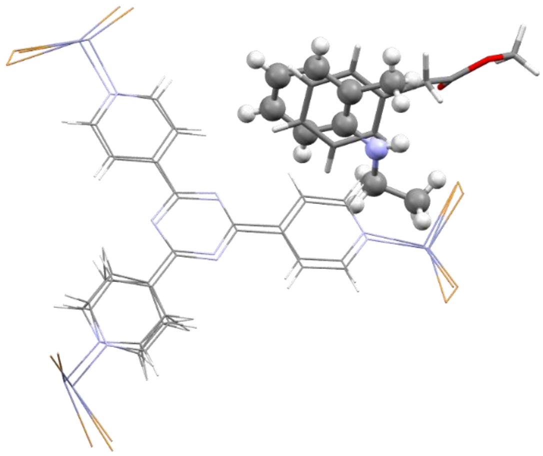

Figure S5. Guest position comparison of the red guest molecules in complexes **2.B** and **2.C** where **B** is displayed as a ball and stick model and **C** is displayed as a capped stick model. The host frameworks are displayed as a wireframe.

Yellow guest molecules

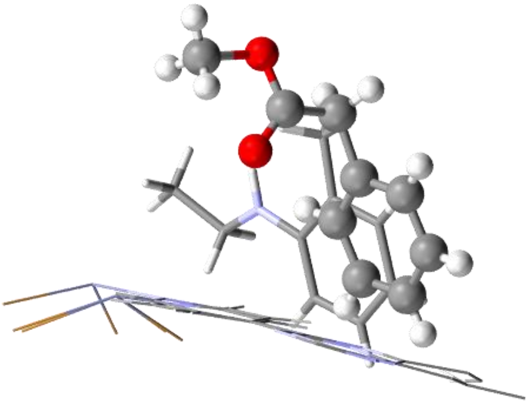

Figure S6. Guest position comparison of the yellow guest molecules in complexes **2.B** and **2.C** where **C** is displayed as a ball and stick model and **B** is displayed as a capped stick model. The host frameworks are displayed as a wireframe.

Blue guest molecules

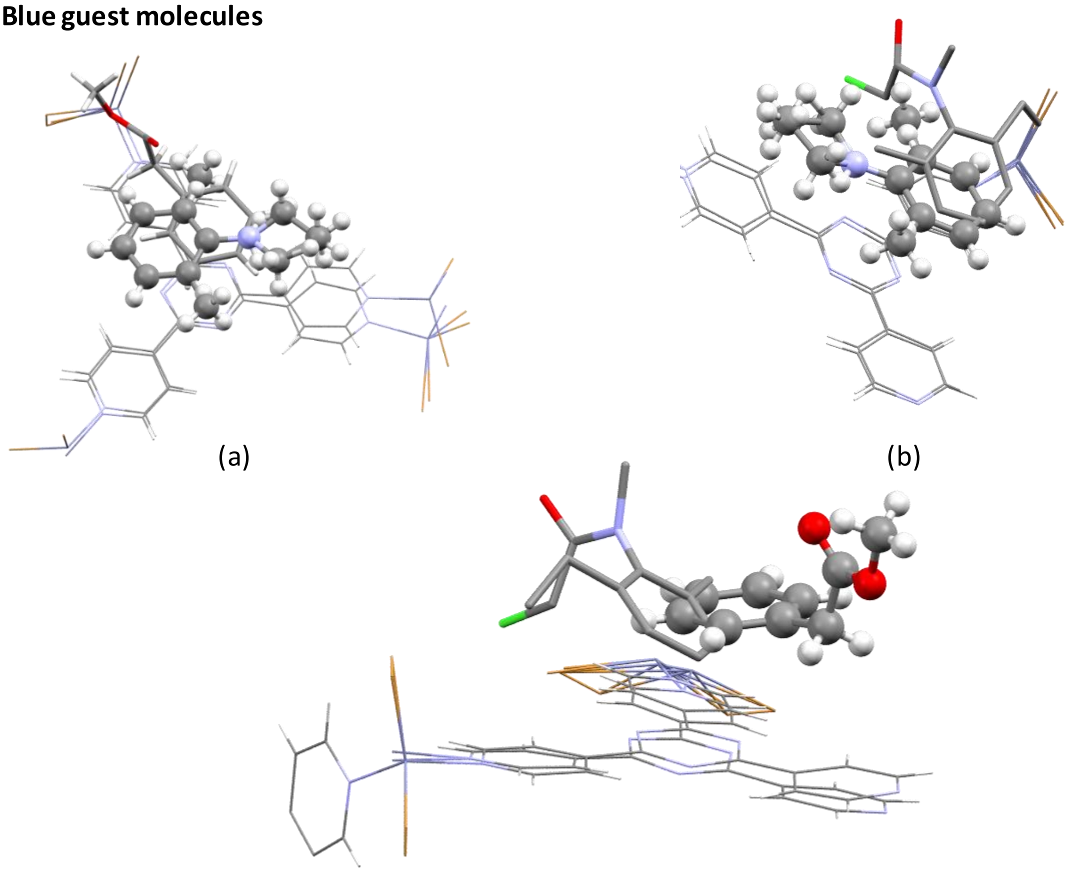

(a) (b) (c)

Figure S7. Guest position comparison of the blue guest molecules in complexes: (a) **2.B** and **2.C** where **B** is displayed as a ball and stick model and **C** is displayed as a capped stick model, (b) **2.B** and **2.E** where **B** is displayed as a ball and stick model and **E** is displayed as a capped stick model and (c) **2.C** and **2.E** where **C** is displayed as a ball and stick model and **E** is displayed as a capped stick model. The host frameworks are displayed as a wireframe.

Green guest molecules

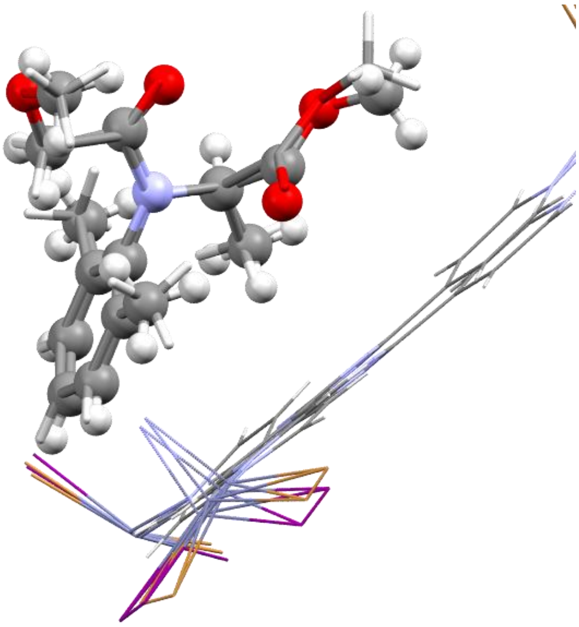

Figure S8. Guest position comparison of the green guest molecules in complexes **1.D** and **2.D** where one molecule of **D** is displayed as a ball and stick model and the other molecule of **D** is displayed as a capped stick model. The host frameworks are displayed as a wireframe.

S6. Metalaxyl-M Inclusion Complexes Intermolecular Interactions

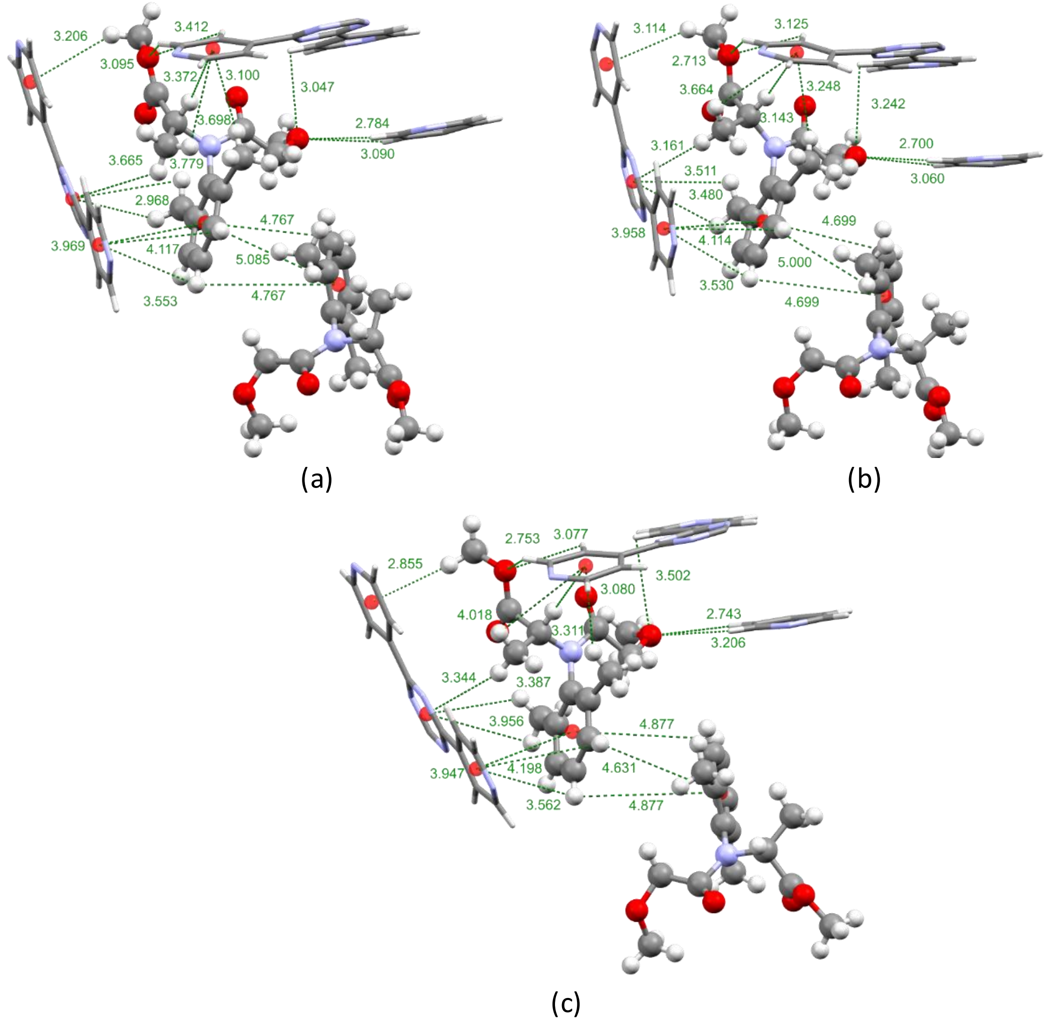

Figure S9. Intermolecular CH···π and π···π interactions formed between the host crystalline sponge framework and guest **D** when **D** was encapsulated into: (a) the host framework **2** at 25 °C, (b) the host framework **2** at 50 °C and (c) the host framework **1** at 50 °C. The host frameworks are displayed as a capped stick model, the guest molecules as a ball and stick model. Centroids are shown as red spheres and the host-guest interactions as green dotted lines. The interactions distances are displayed in angstroms.

S7. General Crystal Structure Refinement Details

Unit cell determinations, absorption corrections and data reduction were performed using the program CrysAlisPro.[1] Crystal structures were solved within the OLEX2 GUI[2] using direct methods in the program SHELXS[3] and refined by full matrix least squares on the basis of F^2 using the program SHELXL.[4] First the successful location and anisotropic refinement of the host MOF framework was always performed then, the guest and solvent molecules were located. Guest and solvent molecules were initially refined without the use of any restraints or constraints, if a stable refinement could not be achieved then the required restraints and constraints were applied. All guest molecules were constrained using the AFIX 66 command to maintain the known structure of the aromatic ring and all non-hydrogen atoms were anisotropically refined unless otherwise specified. Hydrogen atoms were refined using a riding model. Guest and solvent molecule occupancies were all refined towards the end of refinement using free variables before being fixed to the values quoted. In some structures it was not possible to assign all of the residual density peaks in a way that makes chemical sense, these peaks belong to heavily disordered guests or solvent molecules and were taken into account by the use of SQUEEZE function within PLATON.[5] The individual refinement details for each inclusion complex are given separately.

S8. Crystallographic Tables and Individual structure refinement details

Encapsulation Complex **2.A** (guest: phenylacetaldehyde)

CCDC: 2046167

One molecule of phenylacetaldehyde was located within the asymmetric unit. The guest molecule shows no disorder and sits in a general position. The occupancy of the guest molecule was refined freely before being assigned to 90%. The DFIX command was employed to maintain realistic bond lengths. The RIGU and SIMU restraints were used to maintain sensible atomic displacement parameters. The bromine atoms Br1 and Br2 attached to Zn1 are disordered over two positions where both disordered parts were refined at 50% occupancy.

At the end of refinement several electron density peaks were not able to be refined in a way that made any chemical sense, these peaks were taken into account by the use of the SQUEEZE function within PLATON.[5] The solvent mask located one significant void within the asymmetric unit of size 851 $Å^3$ containing 207 electrons.

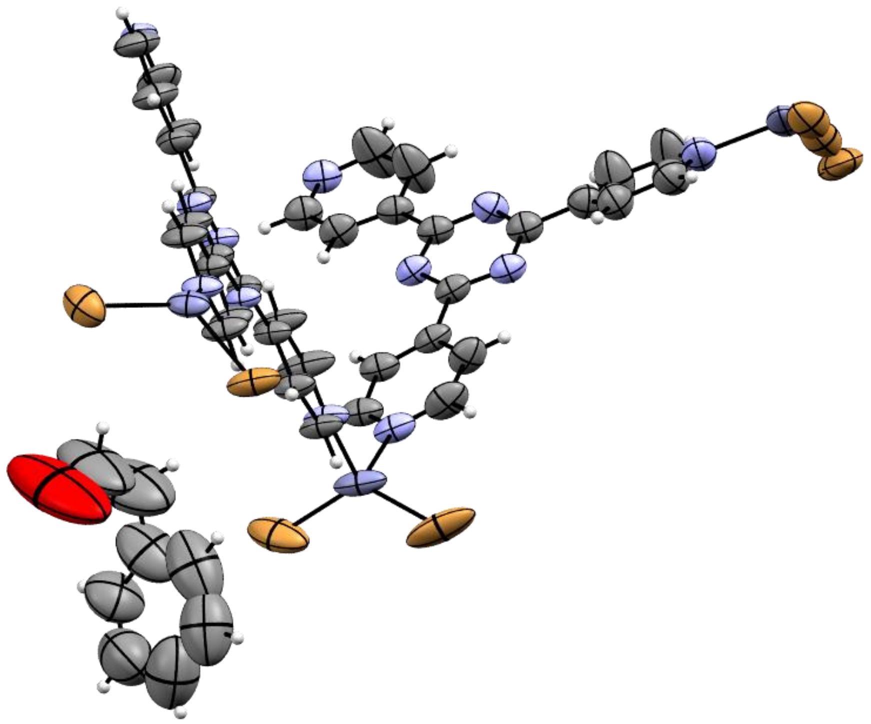

Figure S10. Asymmetric unit of inclusion complex **2.A**.

Table S3. Crystallographic details for inclusion complex **2.A**

Identification code	xstr1222
Empirical formula	$C_{43.2}H_{31.2}Br_6N_{12}O_{0.9}Zn_3$
Formula weight	1408.37
Temperature/K	150(1)
Crystal system	monoclinic
Space group	C2/c
a/Å	34.0605(3)
b/Å	14.72860(14)
c/Å	31.7764(4)
α/°	90
β/°	102.8194(10)
γ/°	90
Volume/Å^3	15543.7(3)
Z	8
ρ_{calc}g/cm^3	1.204
μ/mm^{-1}	4.937
F(000)	5453
Crystal size/mm^3	0.222 × 0.157 × 0.11
Radiation	Cu Kα (λ = 1.54184)
2Θ range for data collection/°	6.918 to 146.028
Index ranges	-42 ≤ h ≤ 42, -18 ≤ k ≤ 16, -38 ≤ l ≤ 39
Reflections collected	126871
Independent reflections	15284 [R_{int} = 0.0350, R_{sigma} = 0.0149]
Data/restraints/parameters	15284/63/601
Goodness-of-fit on F^2	1.040
Final R indexes [I>=2σ (I)]	R_1 = 0.0537, wR_2 = 0.1338
Final R indexes [all data]	R_1 = 0.0618, wR_2 = 0.1410
Largest diff. peak/hole / e Å^{-3}	0.73/-1.01

Encapsulation Complex **2.B** (guest: N-ethyl-*o*-toluidine)

CCDC: 2046400

Four crystallographically independent molecules of N-ethyl-*o*-toluidine were located within the asymmetric unit. Three of these guests sit in general positions and their occupancies were refined freely at 100%. The fourth guest molecule occupies a site with a centre of inversion positioned next to the carbon atom C72, this means the guest molecule is disordered over two positions related by a centre of inversion and each position was refined with 50% occupancy. Hydrogen atoms were not refined on this guest molecule as the hydrogen atoms attached to C72 where not able to be located in the difference Fourier map, this is likely partly due to the close proximity to a centre of inversion. The DFIX restraint was applied to maintain appropriate bond lengths and the restraints RIGU and SIMU were employed to maintain sensible atomic displacement parameters as well as an EADP constraint to constrain the atomic displacement parameters to similar values. Another guest molecule displays disorder, the carbon atoms C37 and C44 are disordered over two positions with occupancies of 70% and 30% for disordered parts 1 and 2 respectively. The DFIX restraint was applied to maintain appropriate bond lengths and the restraints RIGU and SIMU were employed to maintain sensible atomic displacement parameters. One pyridine ring of the TPT linker of the host framework also displays positional disorder where the ring is disordered over two positions with the atoms C11 and N5 in common, the occupancies of the disordered rings were refined freely before being assigned to 50% each. The FLAT and DFIX restraints were used to model the disorder as well as an EADP constraint to constrain the atomic displacement parameters to similar values. The bromine atom Br1 attached to Zn1 is disordered over two positions where both disordered parts were refined at 50% occupancy.

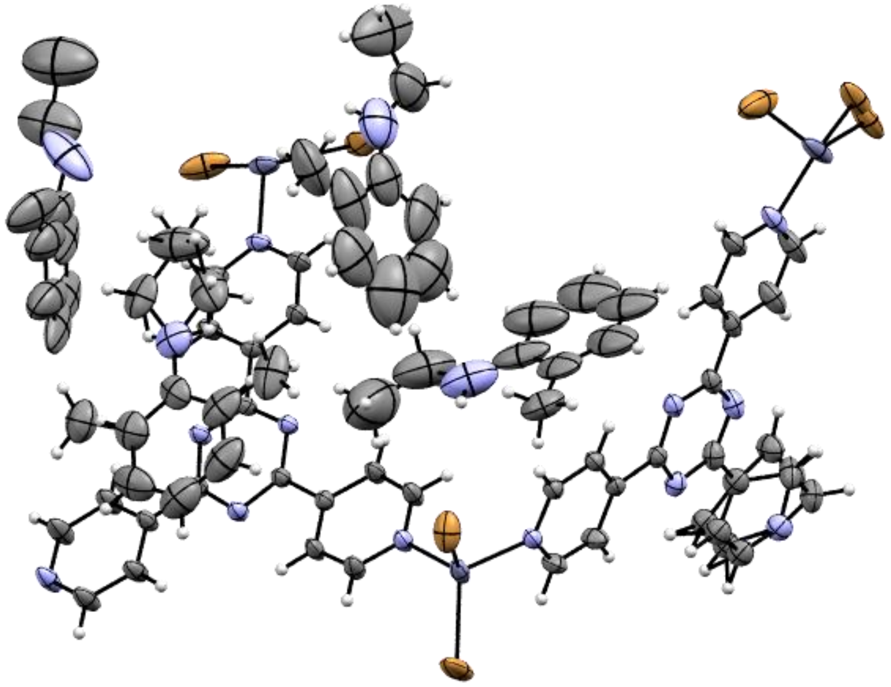

Figure S11. Asymmetric unit of inclusion complex **2.B**

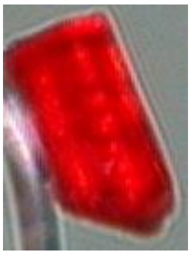

Figure S12. Crystal of complex **2.B**.

Table S4. Crystallographic details for inclusion complex **2.B**

Identification code	xstr1221
Empirical formula	$C_{67.5}H_{63}Br_6N_{15.5}Zn_3$
Formula weight	1766.90
Temperature/K	150.1(4)
Crystal system	monoclinic
Space group	C2/c
a/Å	33.6842(2)
b/Å	14.69785(9)
c/Å	31.54776(19)
α/°	90
β/°	101.2968(6)
γ/°	90
Volume/Å^3	15316.25(16)
Z	8
ρ_{calc}g/cm^3	1.532
μ/mm^{-1}	5.143
F(000)	7012.0
Crystal size/mm^3	0.217 × 0.139 × 0.107
Radiation	Cu Kα (λ = 1.54184)
2Θ range for data collection/°	6.964 to 145.614
Index ranges	-41 ≤ h ≤ 41, -18 ≤ k ≤ 17, -38 ≤ l ≤ 38
Reflections collected	139453
Independent reflections	15061 [R_{int} = 0.0332, R_{sigma} = 0.0118]
Data/restraints/parameters	15061/318/854
Goodness-of-fit on F^2	1.039
Final R indexes [I>=2σ (I)]	R_1 = 0.0659, wR_2 = 0.1776
Final R indexes [all data]	R_1 = 0.0690, wR_2 = 0.1808
Largest diff. peak/hole / e Å^{-3}	1.90/-1.21

Encapsulation Complex **2.C** (guest: Methyl phenylacetate)

CCDC: 2046168

Four molecules of methyl phenylacetate were located within the asymmetric unit. The complete structures of the four guest molecules were fully refined with occupancies of 50%, 52%, 58% and 59%. All occupancies were refined using free variables. One of the fully refined guest molecules displays disorder, specifically the oxygen atom O6 is disordered over two positions with an occupancy of 25% each. The FLAT command was used to ensure C61 remains flat with the phenyl ring. The DFIX restraint has been used to maintain realistic bond lengths and the RIGU and SIMU restraints used to maintain appropriate atomic displacement parameters. The bromine atoms Br1 and Br2 attached to Zn1 also Br5 and Br6 attached to Zn3 are disordered over two positions where both disordered parts were refined at 50% occupancy.

At the end of structural refinement not all of the residual electron density peaks were able to be assigned in a way that made any chemical sense. These peaks were accounted for by the use of the SQUEEZE function within PLATON.[5] One void was located within the asymmetric unit of size 66 Å^3 containing 12 electrons.

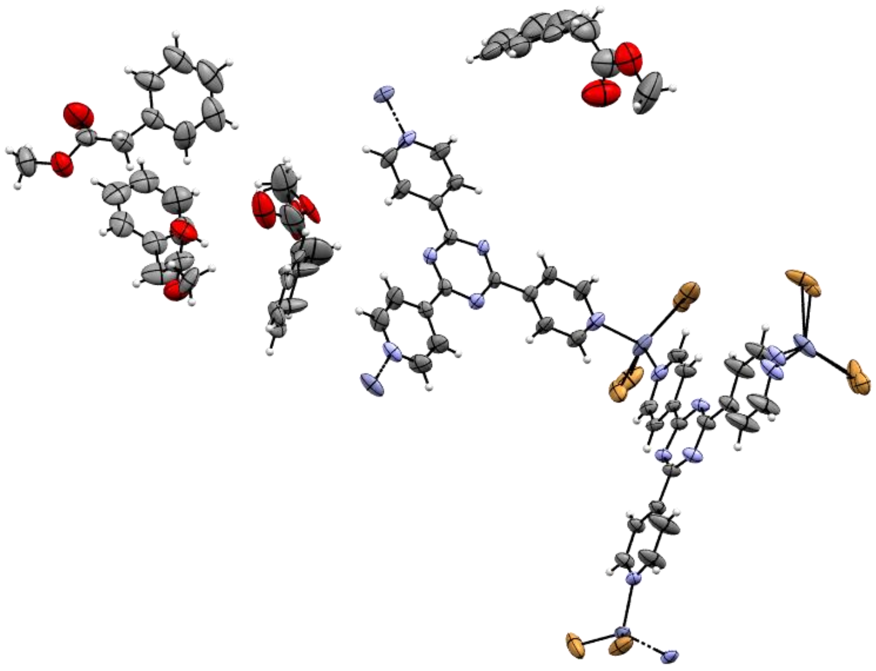

Figure S13. Asymmetric unit of inclusion complex **2.C**

Table S5. Crystallographic details for inclusion complex **2.C**

Identification code	xstr1231
Empirical formula	$C_{55.67}H_{45.86}Br_6N_{12}O_{4.37}Zn_3$
Formula weight	1628.52
Temperature/K	150(1)
Crystal system	monoclinic
Space group	C2/c
a/Å	34.3692(3)
b/Å	14.54887(11)
c/Å	31.5103(3)
α/°	90
β/°	102.9409(8)
γ/°	90
Volume/Å^3	15356.0(2)
Z	8
ρ_{calc}g/cm^3	1.409
μ/mm^{-1}	5.111
F(000)	6391.0
Crystal size/mm^3	0.191 × 0.179 × 0.128
Radiation	Cu Kα (λ = 1.54184)
2Θ range for data collection/°	7.454 to 145.388
Index ranges	-42 ≤ h ≤ 42, -17 ≤ k ≤ 16, -39 ≤ l ≤ 39
Reflections collected	139714
Independent reflections	15087 [R_{int} = 0.0430, R_{sigma} = 0.0169]
Data/restraints/parameters	15087/460/919
Goodness-of-fit on F^2	1.104
Final R indexes [I>=2σ (I)]	R_1 = 0.0987, wR_2 = 0.2420
Final R indexes [all data]	R_1 = 0.1027, wR_2 = 0.2443
Largest diff. peak/hole / e Å^{-3}	1.65/-1.23

Encapsulation Complex **1.D** (guest: Metalaxyl-M)

CCDC: 2046169

One molecule of metalaxyl-M was located and refined within the asymmetric unit. The guest sits in a general position. The guest occupancy was refined freely at 52%. One molecule of chloroform was also located and refined with an occupancy of 25%, the chloroform molecule displayed no signs of disorder. DFIX and SADI restraints were used to maintain realistic bond lengths, RIGU and SIMU restraints were also employed to maintain appropriate atomic displacement parameters and the FLAT restraint was also employed to keep N13 flat with the phenyl ring. Two EADP constraints were used on two of the pyridine rings of the TPT linker molecule of the host framework to constrain the atomic displacement parameters to similar values. One ZnI_2 comprised of the atoms Zn1, I1 and I2 shows disorder over two positions with both disordered parts refined at 50% occupancy. The iodine atoms I3, I5 and I6 all also show positional disorder over two positions where each disordered part has been refined at 50% occupancy.

The SQUEEZE function was used to take into account of electron density peaks that could not be assigned in a way that makes any chemical sense. One significant void was found with a size of 491 Å^3 which contains 44 electrons.

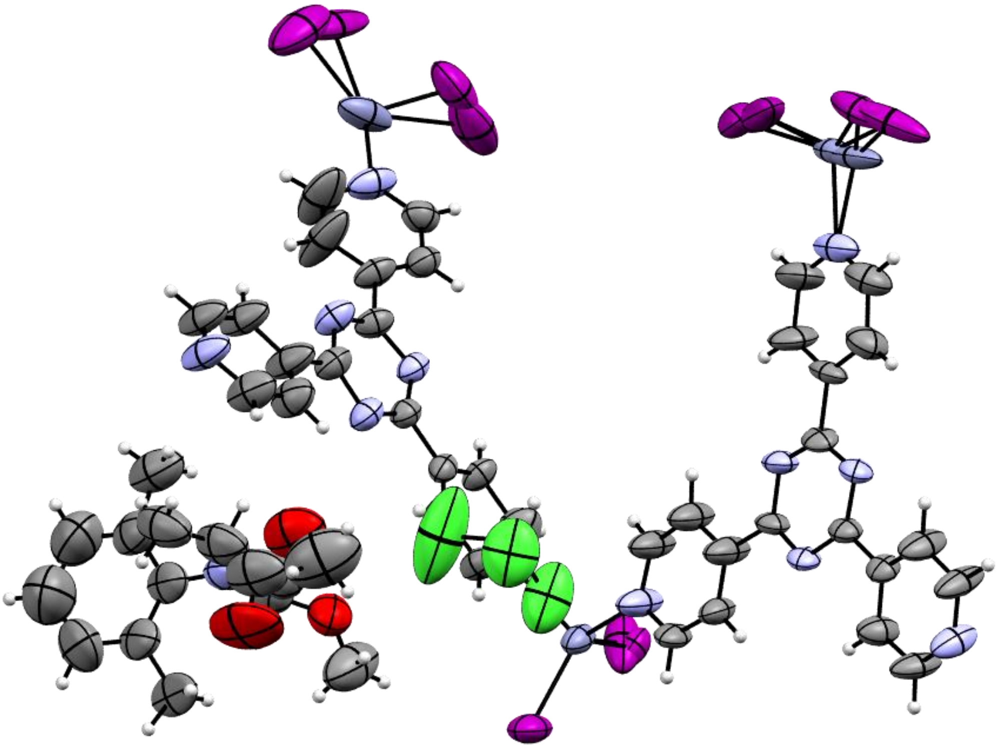

Figure S14. Asymmetric unit of inclusion complex **1.D**

Table S6. Crystallographic details for inclusion complex **1.D**

Identification code	xstr1139
Empirical formula	$C_{44.1}H_{35.24}Cl_{0.75}I_6N_{12.52}O_{2.09}Zn_3$
Formula weight	1758.16
Temperature/K	150(1)
Crystal system	monoclinic
Space group	C2/c
a/Å	35.2951(8)
b/Å	14.9632(2)
c/Å	31.4854(7)
α/°	90
β/°	102.151(2)
γ/°	90
Volume/Å^3	16255.8(6)
Z	8
ρ_{calc}g/cm^3	1.437
μ/mm^{-1}	19.417
F(000)	6600.0
Crystal size/mm^3	0.15 × 0.125 × 0.12
Radiation	Cu Kα (λ = 1.54184)
2Θ range for data collection/°	7.268 to 145.612
Index ranges	-40 ≤ h ≤ 43, -18 ≤ k ≤ 12, -38 ≤ l ≤ 36
Reflections collected	31377
Independent reflections	15598 [R_{int} = 0.0450, R_{sigma} = 0.0454]
Data/restraints/parameters	15598/408/741
Goodness-of-fit on F^2	1.033
Final R indexes [I>=2σ (I)]	R_1 = 0.0991, wR_2 = 0.2519
Final R indexes [all data]	R_1 = 0.1244, wR_2 = 0.2737
Largest diff. peak/hole / e Å^{-3}	1.33/-1.50

Encapsulation Complex **2.D** (guest: Metalaxyl-M)

CCDC: 2046172

One molecule of metalaxyl-M and one molecule of chloroform was located and refined within the asymmetric unit of the inclusion complex **2.D**. The occupancies of both molecules were calculated freely at 58% and 30% respectively. Neither the molecule of metalaxyl-M or the molecule of chloroform displayed any disorder. DFIX and SADI restraints were employed to maintain realistic bond lengths, RIGU and SIMU restraints were used to maintain suitable atomic displacement parameters. The FLAT restraint was used to keep the atom N13 flat with the phenyl ring of metalaxyl-M. All three $ZnBr_2$ substituents of the host framework are disordered over two positions, two of the $ZnBr_2$ substituents were refined with 50% occupancy for each disordered part. The $ZnBr_2$ substituent with the atoms Zn3, Br5 and Br6 was refined with occupancies of 60% and 40% for parts 1 and 2 respectively.

At the end of refinement several residual electron density peaks were unable to be assigned in a way that would make any chemical sense, these were taken into account using the SQUEEZE function of PLATON.[5] One significant void was found of size 509 Å^3 containing 109 electrons.

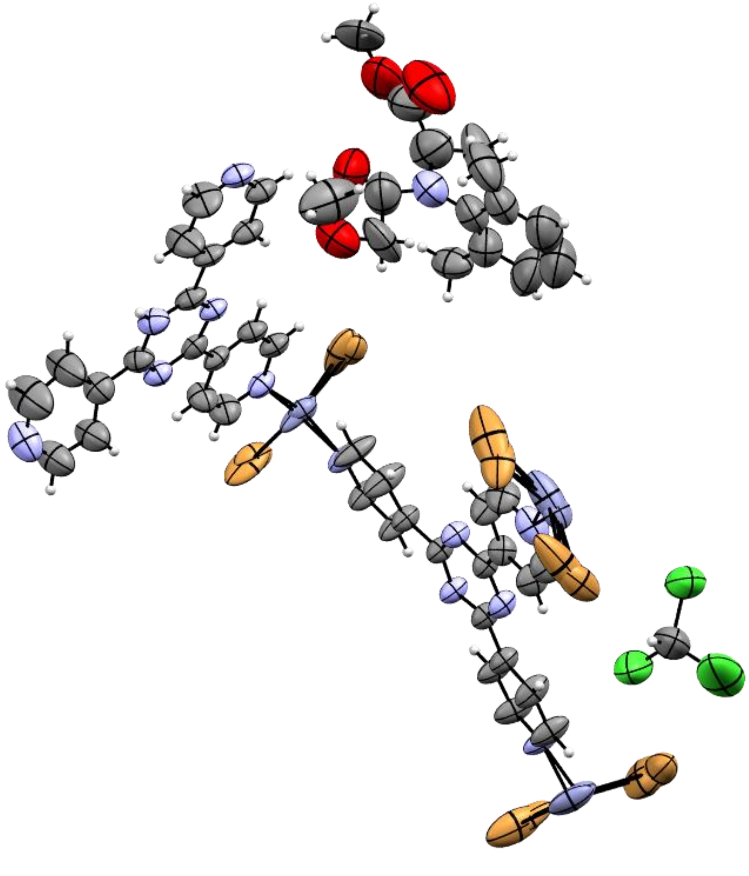

Figure S15. Asymmetric unit of inclusion complex **2.D**

Table S7. Crystallographic details for inclusion complex **2.D**

Identification code	xstr1119
Empirical formula	$C_{45}H_{36.48}Br_6Cl_{0.9}N_{12.58}O_{2.32}Zn_3$
Formula weight	1498.06
Temperature/K	150(1)
Crystal system	monoclinic
Space group	C2/c
a/Å	34.1816(5)
b/Å	14.71740(15)
c/Å	31.7056(5)
α/°	90
β/°	102.2930(14)
γ/°	90
Volume/Å^3	15584.2(4)
Z	8
ρ_{calc}g/cm^3	1.277
μ/mm^{-1}	5.248
F(000)	5827.0
Crystal size/mm^3	0.55 × 0.27 × 0.16
Radiation	Cu Kα (λ = 1.54184)
2Θ range for data collection/°	7.378 to 145.262
Index ranges	$-35 \le h \le 42$, $-17 \le k \le 18$, $-36 \le l \le 39$
Reflections collected	56087
Independent reflections	15120 [R_{int} = 0.0279, R_{sigma} = 0.0195]
Data/restraints/parameters	15120/200/804
Goodness-of-fit on F^2	1.045
Final R indexes [I>=2σ (I)]	R_1 = 0.0816, wR_2 = 0.2235
Final R indexes [all data]	R_1 = 0.0850, wR_2 = 0.2261
Largest diff. peak/hole / e Å^{-3}	0.72/-0.66

Encapsulation Complex **1.E** (guest: S-Metolachlor)

CCDC: 2046170

One molecule of S-metolachlor was located and refined within the asymmetric unit of the inclusion complex **1.E**. The occupancy of the guest molecule was freely refined at 54% and the guest molecule sits in a general position. The molecule of S-metolachlor displays significant disorder where the chloroacetamide is disordered over two positions of the phenyl ring, both disordered parts were refined to 27% occupancy. The nitrogen atoms of the disordered chloroacetamide groups (N12A and N12B) share the same position as the carbon atoms C38B and C49A which represent the disordered ethyl and methyl groups respectively. The second carbon atom of the disordered ethyl group (C37B) and the second methyl group carbon atom (C49B) were unable to be located and refined (Figure S14). As the guest molecule is incomplete the hydrogen atoms of the guest were not modelled. DFIX, RIGU and SIMU restraints were employed to ensure realistic bond lengths and suitable atomic displacement parameters. The iodine atom I1 attached to Zn1 is disordered over two positions where the disordered parts were refined at 66% and 34% occupancy for parts 1 and 2 respectively. The iodine atoms I3 and I4 attached to Zn2 are disordered over two positions where the disordered parts were refined at 54% and 46% occupancy for parts 1 and 2 respectively.

Several electron density peaks remained at the end of structural refinement that could not be assigned in a way that made any chemical sense, these peaks were taken into account by the use of the SQUEEZE function within PLATON.[5] One void was located within the asymmetric unit of size 640 Å^3 which contains 166 electrons.

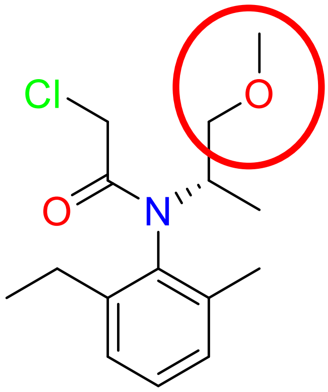

Figure S16. A structure diagram of S-metolachlor with the atoms that could not be located in the crystal structure of inclusion complex **1.E** identified within red circles.

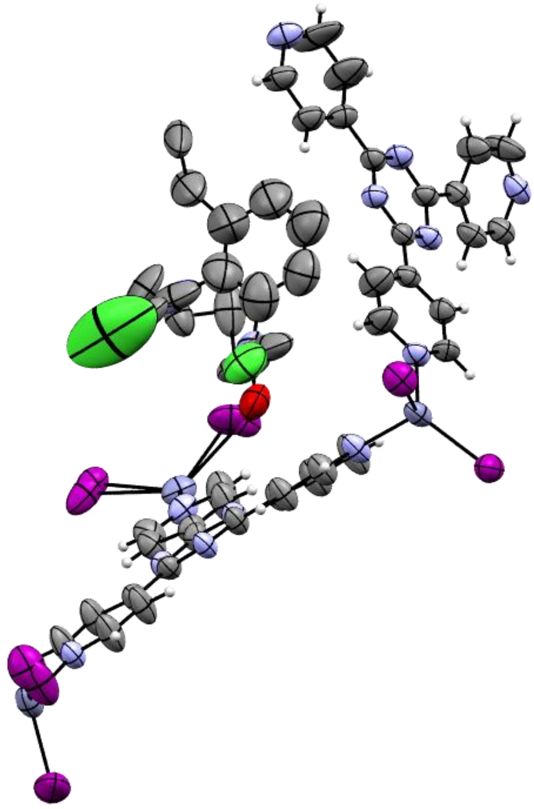

Figure S17. Asymmetric unit of inclusion complex **1.E**

Table S8. Crystallographic details for inclusion complex **1.E**

Identification code	xstr1037
Empirical formula	$C_{42.49}H_{24}Cl_{0.54}I_6N_{12.54}O_{0.54}Zn_3$
Formula weight	1695.57
Temperature/K	150(1)
Crystal system	monoclinic
Space group	C2/c
a/Å	36.2611(10)
b/Å	14.7044(3)
c/Å	31.3139(8)
α/°	90
β/°	102.627(2)
γ/°	90
Volume/Å^3	16292.7(7)
Z	8
ρ_{calc}g/cm^3	1.382
μ/mm^{-1}	19.278
F(000)	6306.0
Crystal size/mm^3	0.247 × 0.109 × 0.092
Radiation	CuKα (λ = 1.54184)
2Θ range for data collection/°	8.34 to 145.762
Index ranges	-44 ≤ h ≤ 44, -18 ≤ k ≤ 17, -38 ≤ l ≤ 36
Reflections collected	56506
Independent reflections	15965 [R_{int} = 0.0569, R_{sigma} = 0.0411]
Data/restraints/parameters	15965/375/740
Goodness-of-fit on F^2	1.054
Final R indexes [I>=2σ (I)]	R_1 = 0.0797, wR_2 = 0.2283
Final R indexes [all data]	R_1 = 0.0915, wR_2 = 0.2408
Largest diff. peak/hole / e Å^{-3}	1.39/-1.88

Encapsulation Complex **2.E** (guest: S-Metolachlor)

CCDC: 2046171

One molecule of S-metolachlor was located and refined within the asymmetric unit of the inclusion complex **2.E**. The guest molecule sits in a general position with an occupancy freely refined at 33%. Four atoms of S-metolachlor could not be located or refined within the crystal structure, three carbon atoms and an oxygen atom, which are identified in Figure S16. As the guest structure is incomplete the hydrogen atoms were not modelled. A FLAT restraint was used on the molecule of S-metolachlor to ensure the atom N13 remains flat with the aromatic plane of the phenyl ring. The restraints RIGU and SIMU were employed on the guest molecule to maintain appropriate atomic displacement parameters and the DFIX restraint used to maintain realistic bond lengths on the guest molecule. All three $ZnBr_2$ substituents of the host framework display disorder over two positions, the $ZnBr_2$ substituent containing atoms Zn1, Br1 and Br2 was refined at 50% occupancy for both disordered parts. The $ZnBr_2$ substituents containing the atoms Zn2, Br3, Br4 and Zn3, Br5, Br6 were refined with occupancies of 60% and 40% for disordered parts 1 and 2 respectively.

Multiple electron density peaks could not be assigned in a way that made any chemical sense by the end of refinement, these peaks were taken into account by the use of the SQUEEZE function within PLATON.[5] One void of size 777 Å^3 was located which contains 180 electrons.

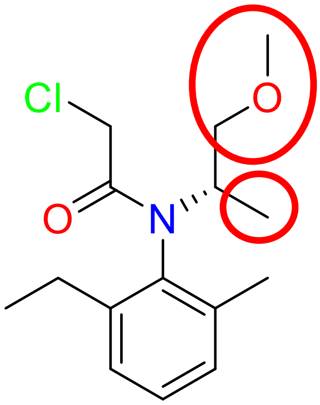

Figure S18. A structure diagram of S-metolachlor with the atoms that could not be located in the crystal structure of inclusion complex **2.E** identified within red circles.

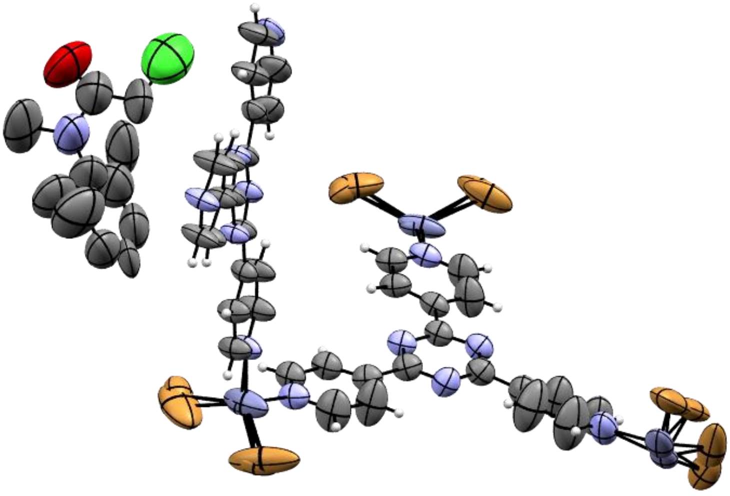

Figure S19. Asymmetric unit of inclusion complex **2.E**

Table S9. Crystallographic details for inclusion complex **2.E**

Identification code	xstr1116
Empirical formula	$C_{40.01}H_{24}Br_6Cl_{0.33}N_{12.33}O_{0.33}Zn_3$
Formula weight	1370.22
Temperature/K	150(1)
Crystal system	monoclinic
Space group	C2/c
a/Å	34.6296(7)
b/Å	14.6765(2)
c/Å	31.9586(6)
α/°	90
β/°	102.581(2)
γ/°	90
Volume/Å^3	15852.7(5)
Z	8
ρ_{calc}g/cm^3	1.148
μ/mm^{-1}	4.925
F(000)	5270.0
Crystal size/mm^3	0.177 × 0.14 × 0.09
Radiation	Cu Kα (λ = 1.54184)
2Θ range for data collection/°	6.922 to 145.424
Index ranges	-42 ≤ h ≤ 21, -12 ≤ k ≤ 18, -36 ≤ l ≤ 39
Reflections collected	30422
Independent reflections	15257 [R_{int} = 0.0240, R_{sigma} = 0.0248]
Data/restraints/parameters	15257/208/718
Goodness-of-fit on F^2	1.066
Final R indexes [I>=2σ (I)]	R_1 = 0.0743, wR_2 = 0.2365
Final R indexes [all data]	R_1 = 0.0814, wR_2 = 0.2467
Largest diff. peak/hole / e Å^{-3}	1.39/-0.63

S9. References

(1) CrysAlisPRO. Agilent Technologies Ltd: Yarnton, Oxfordshire, England 2014.

(2) Dolomanov, O. V; Bourhis, L. J.; Gildea, R. J.; Howard, J. A. K.; Puschmann, H. OLEX2: A Complete Structure Solution, Refinement and Analysis Program. *J. Appl. Crystallogr.* **2009**, *42*, 339–341.

(3) Sheldrick, G. M. A Short History of SHELX. *Acta Crystallogr. Sect. A Found. Crystallogr.* **2008**, *64*, 112–122.

(4) Sheldrick, G. M. Crystal Structure Refinement with SHELXL. *Acta Crystallogr. Sect. C* **2015**, *71*, 3–8.

(5) Spek, A. L. PLATON SQUEEZE: A Tool for the Calculation of the Disordered Solvent Contribution to the Calculated Structure Factors. *Acta Crystallogr. Sect. C Struct. Chem.* **2015**, *71*, 9–18.
